# Metabolomic Profiling of *Floccularia luteovirens* from Different Geographical Regions Proposes a Novel Perspective on Their Antioxidative Activities

**DOI:** 10.3390/antiox13050620

**Published:** 2024-05-20

**Authors:** Chuyu Tang, Yuejun Fan, Tao Wang, Jie Wang, Mengjun Xiao, Min He, Xiyun Chang, Yuling Li, Xiuzhang Li

**Affiliations:** 1 State Key Laboratory of Plateau Ecology and Agriculture, Qinghai Academy of Animal and Veterinary Sciences, Qinghai University, Xining 810016, China; chuyutang0410@163.com (C.T.); fanyuejun_79@163.com (Y.F.); 13085500761@163.com (T.W.); 15574237597@163.com (M.X.); himi1228@163.com (M.H.); 2 State Key Laboratory for Conservation and Utilization of Bio-Resources in Yunnan, Yunnan University, Kunming 650091, China; wangjie1@stu.ynu.edu.cn; 3 Qinghai Institute of Health Sciences, Xining 810016, China; 15909715156@163.com

**Keywords:** *Floccularia luteovirens*, antioxidant capacity, flavonoids, metabolites, altitude

## Abstract

*Floccularia luteovirens*, an endemic resource of the Tibetan Plateau, possesses significant medicinal and ecological values. However, the understanding of antioxidant capacity and metabolic profiling of *F. luteovirens* from diverse regions remains elusive due to limited resources. Therefore, to comprehensively comprehend the antioxidant capacity and metabolite diversity of *F. luteovirens*, we conducted a rounded analysis of its antioxidant capacity from three distinct regions using both untargeted and targeted metabolomics. Determination of antioxidant indices, such as ferric ion-reducing antioxidant power (FRAP), total phenolic content (TPC), and flavonoid content (FC), revealed the robust antioxidant capacity of *F. luteovirens*. QL *F. luteovirens* (QLFL) exhibited no significant difference compared to ZD *F. luteovirens* (ZDFL); however, both were significantly distinct from XH *F. luteovirens* (XHFL) across multiple indices. Furthermore, a positive correlation was observed between FRAP and flavonoid content. A total of 5782 metabolites were identified and chemically classified. Metabolites of *F. luteovirens* varied significantly at different regions and eight key differential metabolites were screened. Phenylalanine, tyrosine and tryptophan biosynthesis, phenylalanine metabolism, and cyanoamino acid metabolism were the main different regulatory pathways. Consequently, the disparities in the antioxidant activity of *F. luteovirens* may primarily be ascribed to the biosynthesis and metabolism of phenylalanine, while vanillic acid could potentially serve as a pivotal metabolite influencing the antioxidative capacity of *F. luteovirens* by targeted metabolomics. These findings enhance our understanding of the composition of *F. luteovirens* and provide valuable resources for its comprehensive utilization and targeted development.

## 1. Introduction

Reactive oxygen species (ROS) are free radicals present in biosystems, which can arise spontaneously during normal cellular energy metabolism or be formed as a result of various environmental factors [1]. Previous studies have demonstrated that imbalanced levels of free radicals in the human body are associated with numerous chronic diseases [2,3]. Oxidative stress arises from the imbalance between the generation of ROS and the elimination of antioxidant mechanisms [4]. A high concentration of free radicals plays a pivotal role in the detriment of human health, primarily inducing cellular apoptosis and peroxidation of cell membranes, thereby contributing to the pathogenesis of neurodegenerative disorders such as Parkinson’s disease and Alzheimer’s disease [5,6]. Endogenous antioxidant defenses can be categorized into non-enzymatic and enzymatic types, including glutathione, ascorbic acid, superoxide dismutase (SOD), catalase (CAT), glutathione peroxidase (GPX), among others [7,8]. Nevertheless, lifestyle modifications have burdened endogenous antioxidants, prompting the quest for safer natural antioxidants to effectively scavenge excessive free radicals. Edible fungi, as specific resources, harbor a substantial quantity of metabolites possessing antioxidant properties, devoid of the side effects and health concerns associated with synthetic antioxidants [9]. Modern pharmacological studies have revealed that numerous edible fungi exhibit a plethora of functionalities, including anti-aging, antioxidant, anti-inflammatory, and neuroprotective properties [10,11]. As a consequence, the consumption of salubrious fungi is the updated option for alleviating human senility and strengthening the immune system.

Edible fungi possess a distinctive flavor and texture due to their rich content of polysaccharides, proteins, polyphenols, vitamins, and other bioactive compounds [12], which are extensively utilized and exploited worldwide nowadays [13]. *Floccularia luteovirens* (Alb. & Schwein.) Pouzar (*F. luteovirens*), commonly named ‘*Armillaria luteovirens* Sacc.’ is a precious ectomycorrhizal fungus growing on the Tibetan Plateau [14]. *F. luteovirens* not only possesses hypoglycemic, antioxidant, and immunomodulatory properties [15], but also plays a crucial role in enhancing species diversity on the Tibetan Plateau and maintaining ecosystem stability within alpine meadows due to its ecological value [16,17]. Given the seasonal dependency and inherent complexities associated with domestication and artificial cultivation, the future trajectory of *F. luteovirens* lies in harnessing its active constituents through strategic exploitation and rational development, aligning with the evolving trends in this field [18]. Currently, many studies have been focused on *F. luteovirens* phenotypes [18], genomes [17], fermentation [19], ecological characteristics, and active components [16]. In terms of active functions, previous studies have demonstrated that *F. luteovirens* polysaccharides have strong antioxidant capacity and also decrease the production of malondialdehyde, thus protecting PC12 cells from hydrogen peroxide-induced oxidative stress [15]. In addition, other studies have revealed the bioactive compounds with antioxidant properties in the ethanol extract of *F. luteovirens* [20]. However, limited research has been conducted to offer a comprehensive overview of the chemical composition and antioxidant properties of *F. luteovirens* across diverse geographical regions.

Following the advancements in multi-omics technology, metabolomics has emerged as a subsequent discipline after genomics and proteomics, providing an optimal and intuitive reflection of dynamic organismal changes during specific processes [21]. Edible fungi possess a wealth of polysaccharides, sterols, lipids, proteins, and other metabolites that hold significant value for extensive scientific exploration [22,23,24]. The antioxidant activity of *F. luteovirens* from three distinct geographical regions was assessed herein. A targeted and untargeted metabolomics approach was employed to analyze the composition and concentration of primary and secondary metabolites in *F. luteovirens*. Additionally, a comparative study was conducted to identify the bioactive components associated with antioxidant activities, thereby providing valuable insights for the further development and utilization of *F. luteovirens*.

## 2. Materials and Methods

### 2.1. Fungal Sample Materials

**Figure 1:**
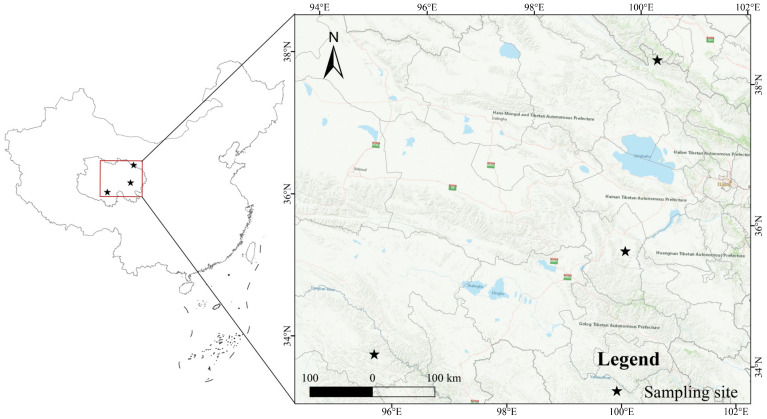
Species distribution points of *F. luteovirens* in different geographic locations. The map was created by ArcGIS (version 10.2, https://www.esri.com/zh-cn/arcgis/, accessed on 24 March 2024).

QL *F. luteovirens*, QLFL (E101.035°, N39.088°; altitude: 3873.5 m), ZD *F. luteovirens*, ZDFL (E95.613°, N33.855°; altitude: 4197.1 m) and XH *F. luteovirens*, XHFL (E99.931°, N35.572°; altitude: 3294.1 m) were purchased from Qinghai Baohuitang Biotechnology Co., Ltd. (Xining, China). They were, respectively, harvested in 2023 (Figure 1). All the fresh samples were stored in a −86 °C ultra-low temperature storage box (Haier Corporation, Qingdao, China) until the metabolomic profiling analysis and the determination of the antioxidant activities.

### 2.2. In Vitro Antioxidant Activity Assay

All kits are purchased from Suzhou Keming Biotechnology Co., Ltd., Suzhou, China.

2,2-diphenyl-1-picrylhydrazyl radical scavenging ability (DPPH•) was determined using a total antioxidant capacity assay kit and conducted as published in previous works, with some modifications [25]. Moreover, 0.1 g of a sample was added to 1 mL of anhydrous sodium acetate–glacial acetic acid solution (extraction solution) for ice bath homogenization, and then 10,000× *g* was centrifuged at 4 °C for 10 min to obtain the sample solution. A total of 50 µL of extracting solution and 950 µL of DPPH solution were added to the blank tubes. A total of 50 µL of the sample solution and 950 µL of DPPH solution were added to the sample tubes. The mixture was mixed and left in the dark at room temperature for 20 min. The absorbance of the reaction solutions was read at 515 nm by spectrophotometry. DPPH• radical scavenging ability was calculated according to Equation (1).

DPPH• radical scavenging ability (%) = [(A_0_ − A_1_)/A_0_] × 100% (1)

where A_0_ was the blank tube absorbance, and A_1_ was the sample tube absorbance.

Hydroxyl free radical scavenging capacity (^•^OH) was operated strictly with the corresponding detection kit instructions. A 0.1 g sample was added to 1 mL of distilled water for ice bath homogenization, and then 10,000× *g* was centrifuged at 4 °C for 10 min to obtain the sample solution. A total of 150 µL of salicylic acid–ethanol solution, 900 µL of distilled water, and 300 µL H_2_O_2_ solution were added to the blank tubes. A total of 150 µL of salicylic acid–ethanol solution, 150 µL of FeSO_4_·7H_2_O, 750 µL of distilled water, and 300 µL of H_2_O_2_ solution were added to the sample control tubes. A total of 150 µL of salicylic acid–ethanol solution, 150 µL of FeSO_4_·7H_2_O, 450 µL of distilled water, 300 µL of H_2_O_2_ solution, and 300 µL of the sample solution were added to the sample tubes. The tubes were kept warm in a 37 °C water bath for 20 min. The absorbance of the reaction solutions was read at 510 nm by spectrophotometry. ^•^OH radical scavenging ability was calculated according to Equation (2).

^•^OH radical scavenging ability (%) = [(A_0_ − A_2_)/A_0_ − A_1_] × 100% (2)

where A_0_ was the sample control tube absorbance, and A_1_ was the blank tube absorbance, A_2_ was the sample tube absorbance.

Superoxide anion radical scavenging capacity (O_2_^•−^) was operated strictly with the corresponding detection kit instructions and conducted as published in previous works, with slight modifications [26]. A 0.1 g sample was added to 1 mL KH_2_PO_4_-K_2_HPO_4_·3H_2_O solution, containing disodium EDTA salt, TritonX-100, and PVP (extraction solution) for ice bath homogenization, and then 10,000× *g* was centrifuged at 4 °C for 10 min to obtain the sample solution. A total of 40 µL of Tris-HCL solution, 100 µL of distilled water, 160 µL ammonium persulfate solution, 200 µL of hydroxylamine hydrochloride solution, 200 µL of P-amino benzene sulfonic acid-acetic acid solution, and 200 µL of alpha naphthylamine acetic acid solution were added to the blank tubes. A total of 40 µL of Tris-HCL solution, 100 µL of the sample solution, 160 µL ammonium persulfate solution, 200 µL of hydroxylamine hydrochloride solution, 200 µL of P-amino benzene sulfonic acid-acetic acid solution, and 200 µL of alpha naphthylamine acetic acid solution were added to the sample tubes. The tubes were kept warm in a 37 °C water bath for 20 min. The absorbance of the reaction solutions was read at 530 nm by spectrophotometry. O_2_^•−^ radical scavenging ability was calculated according to Equation (3).

O_2_^•−^ radical scavenging ability (%) = [(A_0_ − A_1_)/A_0_] × 100% (3)

where A_0_ was the blank tube absorbance, and A_1_ was the sample tube absorbance.

Glutathione peroxidase (GSH-Px) was operated strictly with the corresponding detection kit instructions. A 0.1 g sample was added to 1 mL of the mixed solution (including K_2_HPO_4_·3H_2_O, KH_2_PO_4_, EDTA solution) for ice bath homogenization, and then 8000× *g* was centrifuged at 4 °C for 10 min to obtain the sample solution. The mixed solution consisted of K_2_HPO_4_·3H_2_O, KH_2_PO_4_, EDTA solution, NADPH, GSH, and GR. A total of 100 µL of the sample solution, 800 µL of the mixed solution, and 100 µL of the tert-butyl hydroperoxide solution were added to the sample tubes. The absorbance of the reaction solutions was read at 340 nm by spectrophotometry at 30 s and 210 s. GSH-Px was calculated according to Equation (4).

GSH-Px (nmol/min/g) = 536 × (A_30s_ − A_210s_)/W] (4)

where A_30S_ was the sample tube absorbance at 30 s, and A_210s_ was the sample tube absorbance at 30 s, W was the sample quality.

Catalase (CAT) was operated strictly with the corresponding detection kit instructions. A 0.1 g sample was added to 1 mL of the mixed solution (including Na_2_HPO_4_·12H_2_O and NaH_2_PO_4_ solution) for ice bath homogenization, and then 8000× *g* was centrifuged at 4 °C for 10 min to obtain the sample solution. CAT working solutions (including H_2_O_2_, Na_2_HPO_4_·12H_2_O, and NaH_2_PO_4_ solution) were kept warm in a 25 °C water bath for 10 min. A total of 35 µL of the sample solution and 1 mL working solutions were added to the sample tube to determine the initial absorbance A_1_ at 240 nm and the absorbance A_2_ after 1 min. CAT was calculated according to Equation (5).

CAT (nmol/min/g) = 678 × (A_1_ − A_2_)/W (5)

where A_1_ was the sample tube absorbance at initial absorbance, and A_2_ was the sample tube absorbance after 1 min, W was the sample quality.

Peroxidase (POD) was operated strictly with the corresponding detection kit instructions. A 0.1 g sample was added to 1 mL of the mixed solution (including Na_2_HPO_4_·12H_2_O and NaH_2_PO_4_ solution) for ice bath homogenization, and then 8000× *g* was centrifuged at 4 °C for 10 min to obtain the sample solution. POD working solutions (including Na_2_HPO_4_·12H_2_O, NaH_2_PO_4_ solution, guaiacol solution, and H_2_O_2_ solution) were kept warm in a 25 °C water bath for 10 min. A total of 50 µL of the sample solution and 950 mL working solutions were added to the sample tube to determine 1 min absorbance A_1_ at 470 nm and the absorbance A_2_ after 2 min. POD was calculated according to Equation (6).

POD (U/g) = 2000 × (A_2_ − A_1_)/W (6)

where A_1_ was the sample tube absorbance at 1 min absorbance, and A_2_ was the sample tube absorbance after 2 min, W was the sample quality.

Superoxide dismutase (SOD) was operated strictly with the corresponding detection kit instructions. A 0.1 g sample was added to 1 mL of the mixed solution (including Na_2_HPO_4_·12H_2_O and NaH_2_PO_4_ solution) for ice bath homogenization, and then 8000× *g* was centrifuged at 4 °C for 10 min to obtain the sample solution. SOD working solutions (including adding 250 µL of WST-8 solution to Na_2_HPO_4_·12H_2_O, NaH_2_PO_4_, DTPA solution) were thoroughly mixed. A total of 50 µL of the sample solution, 50 µL xanthine oxidase solution, 800 µL of SOD working solutions, and 100 µL of hypoxanthine solution were added to the sample tubes. A total of 50 µL of distilled water, 50 µL xanthine oxidase solution, 800 µL of SOD working solutions, and 100 µL of hypoxanthine solution were added to the sample control tubes. All tubes were left to stand at room temperature for 30 min. The absorbance of the reaction solutions was read at 450 nm by spectrophotometry, and SOD was calculated according to Equations (7) and (8).

Ax = [(A_1_ − A_2_)/A_1_] (7)

SOD (U/g) = [A_X_/(1 − A_X_) × V_t_]/(W × V_s_/V_t_) (8)

where A_1_ was the sample control tube absorbance, A_2_ was the sample tube absorbance, V_t_ was the total volume of the reaction system, W was the sample quality, and V_s_. was the sample volume added to the reaction system.

Ascorbate peroxidase (APX) was operated strictly with the corresponding detection kit instructions. A 0.1 g sample was added to 1 mL of the mixed solution (including K_2_HPO_4_·3H_2_O, KH_2_PO_4_, PVP solution) for ice bath homogenization, and then 13,000× *g* was centrifuged at 4 °C for 20 min to obtain the sample solution. A total of 100 µL of the sample solution, 700 µL of the mixed solution, 100 µL of ascorbic acid solution, and 100 µL of H_2_O_2_ solution were added to the sample tubes. The absorbance of the reaction solutions was read at 290 nm by spectrophotometry at 10 s and 130 s. APX was calculated according to Equation (9).

APX (nmol/min/g) = 1786 × (A_10s_ − A_130s_)/W] (9)

where A_10S_ was the sample tube absorbance at 10 s, A_130s_ was the sample tube absorbance at 130 s, and W was the sample quality.

Glutathione reductase (GR) was operated strictly with the corresponding detection kit instructions. A 0.1 g sample was added to 1 mL of the mixed solution (including K_2_HPO_4_·3H_2_O, KH_2_PO_4_, and bovine serum albumin solution) for ice bath homogenization, and then 8000× *g* was centrifuged at 4 °C for 15 min to obtain the sample solution. A total of 50 µL of GSSG solution, 100 µL of NADPH solution, 750 µL of the mixed solution, and 100 µL of the sample solution were added to the sample tubes. The absorbance of the reaction solutions was read at 340 nm by spectrophotometry at initial absorbance and 180 s. GR was calculated according to Equation (10).

GR (nmol/min/g) = 536 × (A_1_ − A_2_)/W] (10)

where A_1_ was the initial absorbance, A_2_ was the sample tube absorbance at 180 s, and W was the sample quality.

Total phenolic content (TPC) was operated strictly with the corresponding detection kit instructions. A 0.1 g sample was added to 2 mL of ethanol solution, extracted by oscillating at 60 °C for 2 h, 10,000× *g* was centrifuged at 25 °C for 10 min to obtain the sample solution and volume it with ethanol solution to 2 mL. A total of 50 µL of the sample solution, 250 µL Na_2_CO_3_ solution, and 700 µL of distilled water were added to the sample control tubes. A total of 50 µL of the sample solution, 250 µL Na_2_CO_3_ solution, 250 µL of Folin phenol solution, and 450 µL of distilled water were added to the sample tubes. The absorbance of the reaction solutions was read at 760 nm by spectrophotometry. The linear relationship was y = 5.615x + 0.0012 (R^2^ = 0.9994), and TPC was calculated according to Equation (11).

TPC (mg/g) = 0.356 × (A_2_ − A_1_ − 0.0012)/W (11)

where A_2_ was the sample tube absorbance, A_1_ was the sample control tube absorbance, and W was the sample quality.

Flavonoid content (FC) was operated strictly with the corresponding detection kit instructions. A 0.02 g sample was added to 2 mL of ethanol solution, extracted by oscillating at 60 °C for 2 h, 10,000× *g* was centrifuged at 25 °C for 10 min to obtain the sample solution. A total of 540 µL of distilled water, 30 µL of sodium nitrite solution, 30 µL of aluminum nitrate solution, and 400 µL of NaOH solution were added to the sample control tubes. A total of 540 µL of the sample solution, 30 µL of sodium nitrite solution, 30 µL of aluminum nitrate solution, and 400 µL of NaOH solution were added to the sample tubes. The absorbance of the reaction solutions was read at 510 nm by spectrophotometry. The linear relationship was y = 5.02x + 0.0007 (R^2^ = 0.9996), and FC was calculated according to Equation (12).

FC (mg/g) = 0.398 × (A_2_ − A_1_ − 0.0007)/W (12)

where A_2_ was the sample tube absorbance, A_1_ was the sample control tube absorbance, and W was the sample quality.

Reduced glutathione (GSH) was operated strictly with the corresponding detection kit instructions. A 0.1 g sample was added to 1 mL metaphosphoric acid solution for ice bath homogenization, and then 8000× *g* was centrifuged at 4 °C for 10 min to obtain the sample solution. A total of 100 µL of distilled water, 700 µL of the mixed solution (including K_2_HPO4·3H_2_O, KH_2_PO_4,_ and EDTA solutions), 200 µL of DTNB, and sodium citrate solution were added to the sample control tubes. A total of 100 µL of the sample solution, 700 µL of the mixed solution, 200 µL of DTNB, and sodium citrate solution were added to the sample tubes. The absorbance of the reaction solutions was read at 412 nm by spectrophotometry. The linear relationship was y = 1.5x, and GSH was calculated according to Equation (13).

GSH (μmol/g) = 0.667 × (A_2_ − A_1_)/W (13)

where A_2_ was the sample tube absorbance, A_1_ was the sample control tube absorbance, and W was the sample quality.

Ascorbic acid (AsA) was operated strictly with the corresponding detection kit instructions. A 0.1 g sample was added to 1 mL acetic acid solution for ice bath homogenization, and then 8000× *g* was centrifuged at 4 °C for 20 min to obtain the sample solution. A total of 200 µL of the sample solution, 60 µL EDTA solution, 100 µL acetic acid solution, 240 µL of fast blue B salt, and 1400 µL of distilled water were added to the sample tubes. A total of 200 µL of acetic acid solution, 60 µL EDTA solution, 100 µL acetic acid solution, 240 µL of fast blue B salt, and 1400 µL of distilled water were added to the sample control tubes. The tubes were kept warm in a 25 °C water bath for 20 min. The absorbance of the reaction solutions was read at 420 nm by spectrophotometry. The linear relationship was y = 0.0088x − 0.018 (R^2^ = 0.9978), and AsA was calculated according to Equation (14).

AsA (μg/g) = 113.63 × (A_2_ − A_1_ + 0.018)/W (14)

where A_2_ was the sample tube absorbance, A_1_ was the sample control tube absorbance, and W was the sample quality.

Ferric ion-reducing antioxidant power (FRAP) was operated strictly with the corresponding detection kit instructions. A 0.1 g sample was added to 1 mL anhydrous sodium acetate–glacial acetic acid solution for ice bath homogenization, and then 10,000× *g* was centrifuged at 4 °C for 10 min to obtain the sample solution. The mixed solution includes anhydrous sodium acetate–glacial acetic acid solution, TPTZ solution, and FeCl_3_·6H_2_O solution. A total of 50 µL of anhydrous sodium acetate–glacial acetic acid and 950 µL mixed solution were added to the sample control tubes. A total of 50 µL of the sample solution and 950 µL mixed solution were added to the sample tubes. The tubes were kept warm in a 25 °C water bath for 20 min. The absorbance of the reaction solutions was read at 593 nm by spectrophotometry. The linear relationship was y = 2.4832x + 0.0134 (R^2^ = 0.9996) and FRAP was calculated according to Equation (15).

FRAP (μmol Trolox/g) = 0.4027 × (A_2_ − A_1_ − 0.0134)/W (15)

where A_2_ was the sample tube absorbance, A_1_ was the sample control tube absorbance, and W was the sample quality.

### 2.3. Untargeted Metabolomics Profiling

#### 2.3.1. Samples of Pretreatment and Metabolite Extraction

Samples stored at −80 °C were thawed at room temperature. Moreover, 30 mg of the sample was added to a 1.5 mL Eppendorf tube with two small steel balls and 600 μL methanol–water (V:V = 7:3, with mixed internal standard, 4 μg/mL). After pre-cooling in a −40 °C refrigerator for 2 min and grinding (60 Hz, 2 min), the ultrasonic extraction was conducted in an ice-water bath for 30 min, followed by static incubation at −40 °C for 10~12 h. Subsequently, low-temperature centrifugation was carried out for 10 min (12,000 rpm, 4 °C), and 150 μL of the supernatant was aspirated using a syringe. After filtration through a 0.22 μm organic phase syringe filter, it was transferred to an LC sample vial and stored at −80 °C until LC-MS analysis was performed. Quality control (QC) samples were prepared by mixing extracts from all samples in equal volumes. The vials were stored at −80 °C until LC-MS analysis.

#### 2.3.2. LC-MS/MS Analysis

An ACQUITY UPLC I-Class plus (Waters Corporation, Milford, CT, USA) coupled with a QE plus (Thermo Fisher Scientific, Shanghai, China) high-resolution tandem mass spectrometer was used to analyze the metabolic profiling in both ESI positive and ESI negative ion modes. An ACQUITY UPLC HSS T3 column (1.8 μm, 2.1 × 100 mm) was employed in both positive and negative modes. The binary gradient elution system consisted of (A) water (containing 0.1% formic acid, *v*/*v*) and (B) acetonitrile (containing 0.1% formic acid, *v*/*v*) and separation was achieved using the following gradients: 0–2 min, 95% A, 5% B; 2–14 min, 95% to 0% A, 5% to 100% B; 14 min, 100% B; 15 min, 100% B; and 15.1–16 min, 95% A, 5% B. Formic acid and acetonitrile were provided by Thermo Fisher Scientific. The flow rate was 0.35 mL/min and the column temperature was 45 °C. All the samples were kept at 10 °C during the analysis. The injection volume was 3 μL.

The mass range was from *m*/*z* 100 to 1000. The resolution was set at 70,000 for the full MS scans and 17,500 for HCD MS/MS scans. The Collision energy was set at 10, 20 and 40 eV. The mass spectrometer operated as follows: spray voltage, 3800 V (+) and 3000 V (−); sheath gas flow rate, 35 arbitrary units; auxiliary gas flow rate, 8 arbitrary units; capillary temperature, 320 °C; aux gas heater temperature, 350 °C; s-lens RF level, 50.

### 2.4. Targeted Metabolomics Profiling

#### 2.4.1. Standard Solutions and Calibration Curves

Stock solutions of the single standard were prepared at 1 mg/mL in MeOH. Primary mixed standard stock solution (MSS)was prepared by mixing the single standard stock solution and diluting it with MeOH-water (7:18, *v*/*v*) to appropriate concentrations. Calibration curves were obtained by diluting the MSS with MeOH-water (7:18, *v*/*v*) to the following final concentrations: 200 ng/mL, 80 ng/mL, 32 ng/mL, 12.80 ng/mL, 5.12 ng/mL, 2.05 ng/mL, 0.82 ng/mL, 0.33 ng/mL, 0.13 ng/mL, 0.05 ng/mL, 0.02 ng/mL (Table S1). The mixed standard of 12.8 ng/mL was selected as STD-QC. All solutions and samples were stored at −80 °C until use.

#### 2.4.2. Samples of Pretreatment and Metabolite Extraction

A total of 600 μL of ice-cold MeOH-water (2:1, *v*/*v*, containing IS) was added into the freeze-dried samples (30 mg), then placed into 2 steel balls and ground with a grinder (60 Hz, 2 min). Afterward, the whole samples were extracted by ultrasonic for 20 min in an ice-water bath and then centrifuged at 4 °C (13,000 rpm) for 10 min before decanting 500 μL of supernatant into sample vials. A total of 400 μL of ice-cold MeOH-water (2:1, *v*/*v*, containing IS) was added to the Residue samples. The samples were extracted by ultrasonic for 20 min in an ice-water bath and then centrifuged at 4 °C (13,000 rpm) for 10 min before decanting 300 μL of supernatant to sample vials. We combined the two supernatants and mixed them well. Then the mixed supernatant (200 μL) was dried under a nitrogen stream and re-dissolved in 200 μL of MeOH-water (7:18, *v*/*v*, containing IS), extracted by ultrasonic for 5 min in an ice-water bath, and then filtered through 0.22 μm organic phase pinhole filter for subsequent UPLC-MS/MS analysis. QC was prepared by mixing aliquots of the samples to create a pooled sample.

#### 2.4.3. UPLC-MS/MS Analysis

Liquid chromatography was performed on an ACQUITY UPLC system (Waters Corp.). A Waters ACQUITY UPLC HSS T3 column (1.8 μm, 2.1 × 100 mm) was used for analysis. The injection volume was 5 μL. The mobile phase A was water, containing 0.1% formic acid, and the mobile phase B was ACN. Gradient conditions were as follows with a 0.3 mL/min flow rate: 0–2 min, 0 B; 2–30 min, 0–50% B; 30–32 min, 50–95% B; 32–34 min, 95% B; 34–34.1 min, 100–0% B; 34.1–35.5 min, 0 B. All the samples were kept at 4 °C during the analysis and the column temperature was set at 40 °C. 

Mass spectrometry was performed on the AB SCIEX API 6500^+^ Qtrap System (AB SCIEX, Framingham, MA, USA), with an electrospray ionization (ESI) source, operating in both positive and negative ion modes. Nitrogen was employed as the collision gas. Additional instrumental parameters were as follows: positive ion mode: CUR: 35 Psi; EP: 10 V; IS: 5500 V; CXP: 10 V; TEM: 500 °C; Gas1: 60 Psi; Gas2: 50 Psi; negative ion mode: CUR: 35 Psi; EP: −10 V; IS: −4500 V; CXP: −20 V; TEM: 500 °C; Gas1: 60 Psi; Gas2: 50 Psi.

Targeted metabolites were analyzed in a scheduled multiple reaction monitoring (SRM) mode. The MRM pairs, declustering potentials (DPs), and collision energies (CEs) were optimized for each analyte. Data acquisitions and further analyses were conducted using Analyst (version 1.7.2). SCIEX OS-MQ software (version 1.6.1.29803) was used to quantify all metabolites.

### 2.5. Data Preprocessing and Statistical Analysis

All quantitative data for in vitro antioxidant capacity determination were consistent with normal distribution and were analyzed with one-way ANOVA performed by IBM SPSS Statistics (version 26.0). For the data of untargeted metabolomics, the original LC-MS data were processed by the software Progenesis QI V2.3 (Nonlinear, Dynamics, Newcastle, UK) for baseline filtering, peak identification, integral, retention time correction, peak alignment, and normalization. Compound identification was based on the precise mass-to-charge ratio (*m*/*z*), secondary fragments, and isotopic distribution using the human metabolome database (HMDB), LIPID MAPS (V2.3), METLIN, and Luming’s self-built databases. Multivariate statistical analysis determined the stability of the entire analysis process using principal component analysis (PCA), followed by orthogonal partial least squares-discriminant analysis (OPLS-DA) was used to differentiate overall differences in metabolic profiles between groups and to find differential metabolites. Variable importance of projection (VIP) values obtained from the OPLS-DA model were used to rank the overall contribution of each variable to group discrimination. A two-tailed Student’s *t*-test was further used to verify whether the metabolites of difference between groups were significant. Criteria for differential metabolite screening were VIP > 1 and *p*-value < 0.05. In addition, volcano maps, clustered heat maps, and Venn were mapped and analyzed via the cloud platform (https://cloud.oebiotech.cn/, accessed on 19 March 2024). Functional annotation and metabolic pathways of the differential metabolites were assessed by the database of the Kyoto Encyclopedia of Genes and Genomes (KEGG, https://www.genome.jp/kegg/, accessed on 21 March 2024). For the targeted metabolomics data, metabolite quantification was performed using triple quadrupole mass spectrometry in selected reaction detection (SRM) mode, and metabolite identification was performed using SCIEX OS-MQ software (version 1.6.1.29803). The peak area of each chromatographic peak represented the relative amount of the corresponding metabolite, and the peak area of the metabolite was brought into the regression equation fitted to the standard curve to obtain the metabolite content. Correlation analysis was performed by ChiPlot (https://www.chiplot.online/, accessed on 29 March 2024). The raw LC-MS data were deposited in the national genomics data center (https://ngdc.cncb.ac.cn/, accessed on 6 February 2024) with the accession number OMIX005829.

## 3. Results

### 3.1. Comparative Analysis of In Vitro Antioxidant Activity

**Figure 2:**
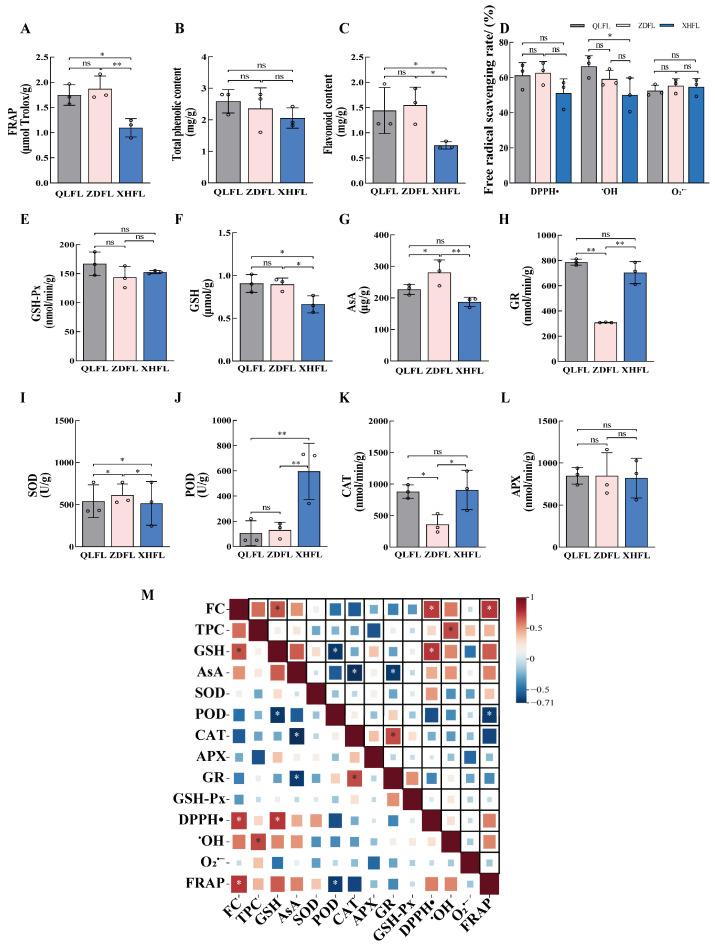
Comparison of antioxidant capacity of *F. luteovirens* from different geographical locations. (**A**) Ferric ion-reducing antioxidant power (FRAP); (**B**) total phenolic content (TPC); (**C**) flavonoid content (FC); (**D**) 2,2-diphenyl-1-picrylhydrazyl radical scavenging ability (DPPH•); hydroxyl free radical scavenging capacity (^•^OH); superoxide anion radical scavenging capacity (O_2_^•−^); (**E**) glutathione peroxidase (GSH-Px); (**F**) reduced glutathione (GSH); (**G**) ascorbic acid (AsA); (**H**) glutathione reductase (GR); (**I**) superoxide dismutase (SOD); (**J**) peroxidase (POD); (**K**) catalase (CAT); (**L**) ascorbate peroxidase (APX); (**M**) Index correlation. “*” indicates a significant difference (*p* < 0.05); “**” indicates an extremely significant difference (*p* < 0.01); “ns” indicates the difference is not significant (*p* > 0.05).

The evaluation of the antioxidant capacity of *F. luteovirens* from different regions can be accomplished by assessing a diverse range of antioxidant substances, including both enzymatic and non-enzymatic components. Through the determination of 14 antioxidant indexes (Figure 2A–L), total phenolic content (TPC) of QLFL, ZDFL, and XHFL were 2.58 ± 0.36 mg/g, 2.35 ± 0.66 mg/g, and 2.05 ± 0.32 mg/g, respectively, while the APX of QLFL, ZDFL, and XHFL reached 848.35 ± 99,848.35 ± 275,821.56 ± 236 nmol/min/g, respectively, and the GSH-Px of QLFL was up to 167.05 ± 20 nmol/min/g but was not significantly different from the other two regions (*p* > 0.05). We revealed no significant difference in APX, GSH-Px, and TPC of *F. luteovirens* from different regions (*p* > 0.05). Notably, the FC in QLFL and ZDFL was 1.44 mg/g and 1.54 mg/g, respectively, whereas the FC content in XHFL amounted to 0.75 mg/g and was consistent with the trend of FRAP results. GSH, FRAP, and the FC of QLFL and ZDFL were significantly higher than those of XHFL (*p* < 0.05). ZDFL had a maximum AsA of 280.66 ug/g, but GR and CAT were significantly lower than the others. Additionally, the determination of SOD and POD revealed that XHFL and ZDFL had the highest enzyme activities. Interestingly, the DPPH•, O_2_^•−^, ^•^OH scavenging capacities of *F. luteovirens* from all three regions exceeded 50% and showed strong scavenging capacities, with no significant difference except for the ^•^OH scavenging capacity of QLFL (*p* > 0.05), which was significantly higher than that of XHFL. We conducted a correlation analysis of antioxidant indexes and discovered that FC was correlated with a significant positive correlation with FRAP, DPPH•, and GSH (Figure 2M). Thus, we speculate that the antioxidant capacity of *F. luteovirens* from different regions may be related to their metabolites, especially flavonoid phenolic metabolites.

### 3.2. Metabolite Profiles of the F. luteovirens

**Figure 3:**
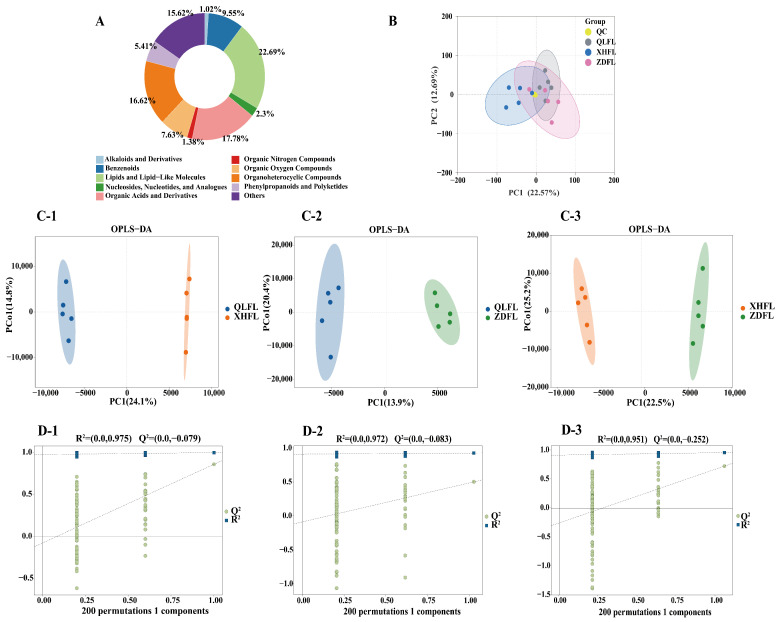
Overview and multivariate statistical analysis of *F. luteovirens*. (**A**) Classification of all measured metabolites; (**B**) principal component analysis of QLFL, XHFL, ZDFL, and QC samples; (**C-1**) plots of OPLS-DA scores in QLFL and XHFL; (**C-2**) plots of OPLS-DA scores in QLFL and ZDFL; (**C-3**) plots of OPLS-DA scores in XHFL and ZDFL; (**D-1**) OPLS permutation test plots of 200 random permutations for QLFL and XHFL; (**D-2**) OPLS permutation test plots of 200 random permutations for QLFL and ZDFL; (**D-3**) OPLS permutation test plots of 200 random permutations for XHFL and ZDFL.

The aforementioned experiments have demonstrated variations in the antioxidant capacity of *F. luteovirens* across different geographical origins. Consequently, a metabolome assessment was employed to elucidate the composition of these compounds comprehensively. Base peak chromatograms (BPCs) of samples are commonly utilized to evaluate the reproducibility between duplicate samples within a cohort. By superimposing the BPC obtained from positive and negative ion detection modes, it becomes evident that the separation between adjacent peaks for each sample is conspicuous, thereby demonstrating the stability and reliability of data collected by the detection system in both positive and negative ion modes (Table S2). Here, we detected a total of 5782 metabolites, including 1312 lipids and lips-like molecules, 1028 organic acids and derivatives, 961 organoheterocyclic compounds, 903 others, 552 benzenoids, 441 organic oxygen compounds, 313 phenylpropanoids and polyketides, 133 nucleosides, nucleotides, and analogs, 80 organic nitrogen compounds, 59 alkaloids and derivatives (Figure 3A). To further investigate the intrinsic differences among the metabolites detected in *F. luteovirens*, we performed principal component analysis (PCA) on the metabolite data. The first main component explained (22.57%) of the total variability while the second principal component accounted for (12.69%) of the total variability of the data set (Figure 3B). Results of the principal component analysis showed clustering within the groups and the partial intersection between the groups of the samples of QLFL, ZDFL, and XHFL. The observed phenomenon can be attributed to the metabolite similarity between QLFL and ZDFL metabolites, while they exhibit significant differences from XHFL.

### 3.3. Screening of Differential Metabolites

In contrast to the PCA model, the OPLS-DA model is a supervised discriminant analysis statistical method, resulting in better classification and prediction. To optimize intergroup separation, OPLS-DA was used for further analysis after the removal of the quality control samples. Within the model, R^2^X (cum) and R^2^Y (cum) represent the explanatory rate of the constructed model for the X and Y matrices, respectively, and the explanatory rate of the constructed model for the X and Y matrices, respectively. Q^2^ denotes the predictive power of the model. We used the OPLS-DA model to compare the metabolite composition of QLFL vs. XHFL (R^2^X = 0.549, R^2^Y = 0.998, Q^2^ = 0.858; Figure 3(C-1)), QLFL vs. ZDFL (R^2^X = 0.558, R^2^Y = 0.989, Q^2^ = 0.541; Figure 3(C-2)), XHFL vs. ZDFL (R^2^X = 0.611, R^2^Y = 0.996, Q^2^ = 0.756; Figure 3(C-3)). All pairwise comparisons calculated higher R^2^X, R^2^Y, and Q^2^ values. OPLS-DA results showed a clear separation of metabolites between *F. luteovirens* from different regions. To avoid overfitting the supervised model during the modeling process, the replacement test was used to test the model to ensure the validity of the model. As the R^2^ and Q^2^ of the stochastic model gradually decreased, it showed that there was no overfitting of the original model (Figure 3(D-1–D-3)), which indicated that the separation of metabolites between groups was statistically significant.

**Figure 4:**
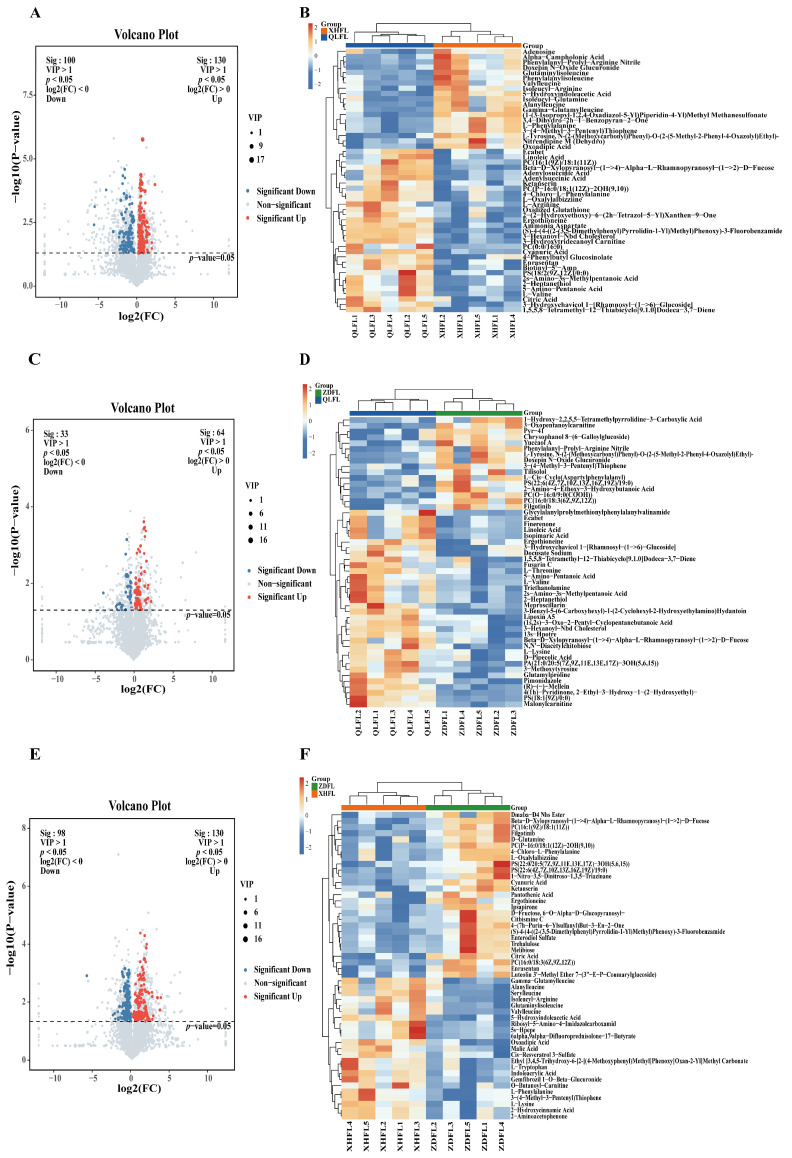
Differential metabolomic analysis from *F. luteovirens*. (**A**) Volcano plots of QLFL vs. XHFL; (**B**) hierarchical clustering of QLFL vs. XHFL; (**C**) volcano plots of QLFL vs. ZDFL; (**D**) hierarchical clustering of QLFL vs. ZDFL; (**E**) volcano plots of XHFL vs. ZDFL; (**F**) hierarchical clustering of XHFL vs. ZDFL.

**Figure 5:**
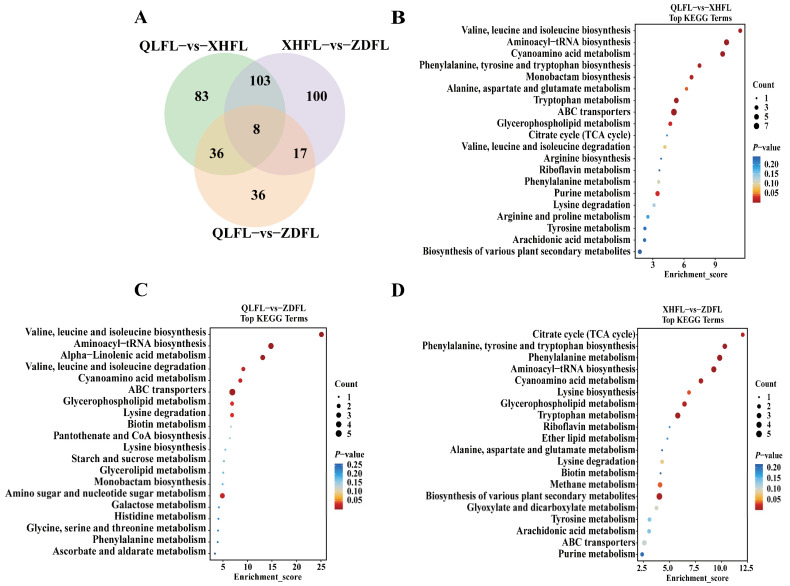
Venn diagram and pathway analysis of differential metabolites. (**A**) Venn diagram of overlapping and unique metabolites between groups; (**B**–**D**) KEGG enrichment of differential metabolites between groups. The horizontal coordinate enrichment score is the enrichment score and the vertical coordinate is the top 20 pathway information. Pathways with larger bubbles contain more differential metabolites, the bubble color changes from blue–red, and their enrichment *p*-value values are smaller and more significant.

The criteria of *p*-value < 0.05 and VIP > 1 were applied to filter out the significant differential metabolites within each pairwise comparison, differential metabolites were illustrated by volcano plots (Figure 4A,C,E) and Venn diagrams (Figure 5A). To more visually demonstrate the differences in metabolite expression among *F. luteovirens* in different regions, hierarchical clustering was performed on the expression of all significant differential metabolites and significant differential metabolites according to the VIP Top50, respectively (Figure 4B,D,F). There were 230 differential metabolites of QLFL vs. XHFL (130 upregulated and 100 downregulated, Figure 4A), 97 differential metabolites of QLFL vs. ZDFL (64 upregulated and 33 downregulated, Figure 4C), and 228 differential metabolites of XHFL vs. ZDFL (130 upregulated and 98 downregulated, Figure 4E). Identified compounds were mainly composed of organic acids and derivatives, lipids, lipid-like molecules, organoheterocyclic compounds, phenylpropanoids, and polyketides, which may be metabolites associated with the different antioxidant activities observed (Figure S1). Furthermore, we identified eight overlapping differential metabolites that serve as potential biomarkers for distinguishing *F. luteovirens* from different regions, suggesting their differential regulation during growth in distinct geographical regions (Table S3).

### 3.4. KEGG Pathway Annotation of the Differential Metabolites

The Kyoto Encyclopedia of Genes and Genomes (KEGG) database (https://www.genome.jp/kegg/, accessed on 21 March 2024) is a primary database that contributes to the comprehension of the mechanisms of metabolic pathway changes in differential samples through pathway enrichment analysis of differential metabolites. Here, we annotated the differential metabolites of each group and categorized them into different pathways. Moreover, 39, 25, and 32 pathways were involved in the differential metabolites of QLFL vs. XHFL, QLFL vs. ZDFL, and ZDFL vs. XHFL, respectively, and the major pathways are shown in the bubble diagrams (Figure 5B–D). Most remarkably, amino acid and carbohydrate metabolism were significantly upregulated in three groups.

**Figure 6:**
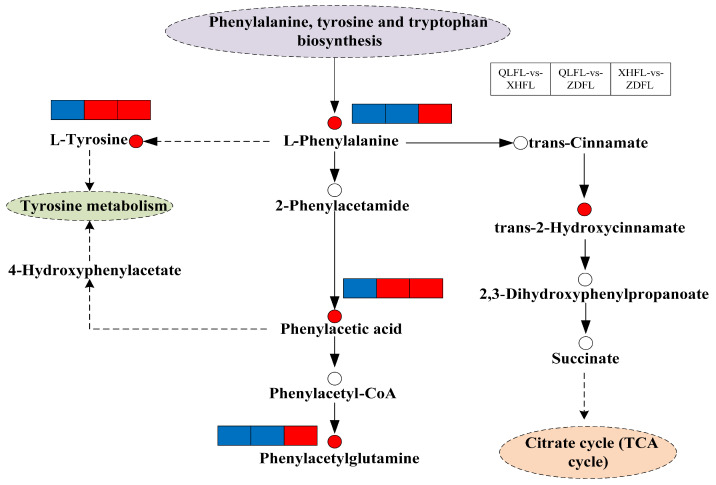
Overview of metabolic pathways mapped to possible regulation of key metabolites in pairwise comparisons of *F. luteovirens* from different regions. Note: Small red circles indicate significant upregulation of metabolites; small empty circles indicate undetected metabolites; small red rectangles indicate significant upregulation of metabolites between groups; small blue rectangles indicate significant downregulation of metabolites between groups; solid arrows represent facilitation, and dotted arrows represent indirect facilitation.

The most notable finding is the significant upregulation of metabolic pathways associated with flavonoid biosynthesis, specifically phenylalanine, tyrosine, and tryptophan biosynthesis, as well as phenylalanine metabolism in the XHFL vs. ZDFL comparison (Figure 5D). Apart from the enrichment of differential metabolites in flavonoid biosynthesis, significantly enriched metabolic pathways in QLFL vs. ZDFL (Figure 5C) and QLFL vs. XHFL (Figure 5B) were valine, leucine, and isoleucine biosynthesis, aminoacyl-tRNA biosynthesis, and cyanoamino acid metabolism. These results indicated that *F. luteovirens* from these three origins may have different metabolite profiles, which may be linked to the antioxidant capacity of *F. luteovirens*. Further, the metabolic pathways of the most relevant overlapping differential metabolites were screened based on KEGG and enrichment maps to facilitate an overview of changes in metabolic regulation (Figure 6). In the phenylalanine, tyrosine, and tryptophan biosynthesis pathways, metabolites such as L-phenylalanine, L-tyrosine, and phenylacetic acid were upregulated as there was speculation that the synthesis and phenylalanine metabolism may be relevant to environmental factors in different regions.

### 3.5. Targeted Metabolomic Analysis

**Figure 7:**
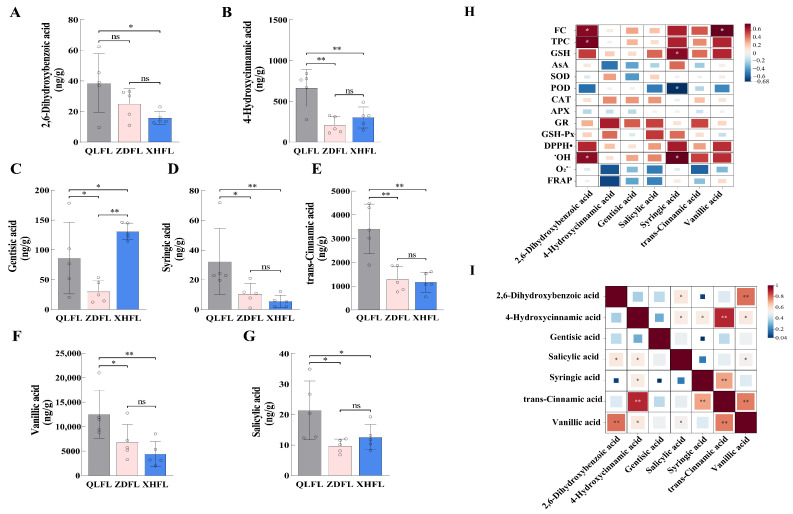
Quantitative and correlation analysis of differential metabolites. (**A**–**G**) Quantitative analysis of differential metabolites; (**H**) Correlation analysis of differential metabolites with indexes of antioxidant capacity; (**I**) Intragroup correlation analysis of differential metabolites. “*” indicates a significant difference (*p* < 0.05); “**” indicates an extremely significant difference (*p* < 0.01); “ns” indicates the difference is not significant (*p* > 0.05).

Based on the determination of antioxidant capacity and untargeted metabolomics analysis, we observed significant differences in the flavonoid content of *F. luteovirens* and indicated that the differential metabolites were potentially enriched in phenylalanine metabolism. Thus, targeted metabolomics of flavonoid phenolics was carried out to validate the above speculation that flavonoid phenolic metabolites influence the antioxidant capacity of *F. luteovirens*. We characterized and quantified 34 flavonoid-phenolic metabolites in *F. luteovirens* (Table S4). Differential metabolites were compared between groups by t-test and fold change, and seven were finally identified as differential metabolites. Except for gentisic acid, which was significantly higher in XHFL than in QLFL and ZDFL, the remaining differential metabolites were highly enriched in QLFL (Figure 7A–G).

Meanwhile, the antioxidant capacity data were correlated with the targeted metabolomics results to explore the relationship between different origins of *F. luteovirens* and antioxidant capacity. We found that 2,6-Dihydroxybenzoic acid was positively correlated with FC, TPC, and ^•^OH, vanillic acid was also significantly correlated with FC, GSH, and ^•^OH was positively correlated with syringic acid, and negatively correlated with POD (Figure 7H). Furthermore, intragroup correlation analysis of the targeted differential metabolites revealed that vanillic acid was highly significantly correlated with 2,6-Dihydroxybenzoic acid and trans-cinnamic acid, and significantly correlated with 4-Hydroxycinnamic acid and salicylic acid. Therefore, we hypothesized that vanillic acid, a phenolic acid, significantly impacts the antioxidant capacity of *F. luteovirens* from various regions (Figure 7I).

## 4. Discussion

Oxidative stress caused by reactive oxygen species produced from diverse sources is a source of pathogenesis for a wide range of diseases [27]. Previous studies have confirmed that mushrooms are resistant to powerful antioxidant functions and that wild mushroom passages contain somewhat higher levels of secondary metabolites than cultivated mushrooms [10,28,29]. *F. luteovirens*, which have economic and ecological value, is widely recognized as a wild medicinal resource by the Qinghai–Tibet Plateau of China [16]. However, research on it as an endemic resource has been limited. In this study, we observed that *F. luteovirens* from different regions have strong antioxidant capacity. We observed no significant difference (*p* > 0.05) in the QLFL, ZDFL, and XHFL, which were determined at 2.58 ± 0.36 mg/g, 2.35 ± 0.66 mg/g, and 2.05 ± 0.32 mg/g respectively, consistent with the observed trends in APX and GSH-Px results. FC of QLFL and ZDFL was determined at 1.44 mg/g and 1.54 mg/g, respectively, which exhibited a significant increase compared to XHFL (*p* < 0.05), consistent with the observed trends in FRAP and GSH results. The maximum AsA value of ZDFL was 280.66 ug/g, but GR was significantly lower than others. SOD, POD, and CAT showed that QLFL, XHFL, and ZDFL had higher enzyme activity. GSH and AsA are non-enzymatic types of antioxidants, which are one of the key components of the ascorbate–glutathione cycle [30]. Here, we observed that QLFL and ZDFL exhibited higher levels of GSH and AsA compared to XHFL, thereby indirectly suggesting their superior antioxidant capacity. GR and APX, as the key enzymes of this cycle, also play important roles in the antioxidant system [31], and GR was the highest in QLFL. Furthermore, we determined the scavenging capacities of DPPH•, O_2_^•−^, and ^•^OH, and found that *F. luteovirens* from different regions could achieve a 50% scavenging rate; we also found no significant difference in TPC. Notably, comparing FC, we found that QLFL and ZDFL were significantly higher than XHFL, while correlation analysis found that FRAP was positively correlated with FC. Flavonoids are a class of metabolites that are widespread in plants and fungi and can perform as electron donors with a variety of effects such as antioxidant, preventing vascular proliferation, anti-inflammatory, and hypoglycemic lipids [32,33]. Combined with many previous studies demonstrating that flavonoid is closely related to antioxidant functions, we further analyzed the metabolic profile of *F. luteovirens* to investigate the relationship between metabolites and antioxidation.

A total of 5782 metabolites of 10 superclasses were identified, including lipids and lipid-like molecules (22.69%), organic acids and derivatives (17.78%), organoheterocyclic compounds (16.62%), benzenoids (9.55%), etc. OPLS-DA indicated that the metabolites contained in the three groups varied considerably. Moreover, 230 metabolites differed significantly between QLFL vs. XHFL, 97 metabolites between QLFL vs. ZDFL, and 228 metabolites between XHFL vs. ZDFL, and these metabolites can be considered biomarkers in *F. luteovirens*. Such comprehensive metabolomics analysis provided a comprehensive view of the metabolic profile of *F. luteovirens* from different regions and helped in the screening of differential metabolites and identification of potential biomarkers. Differential metabolites of the three groups were analyzed in detail. It was noticed that phenylalanine, tyrosine, and tryptophan biosynthesis pathways and phenylalanine metabolism might be the main reasons for the differences in antioxidant activity. Flavonoid biosynthesis initiates with phenylalanine, which is catalyzed by the core phenylalanine-related biosynthetic genes phenylalanine ammonia-lyase (PAL), cinnamate 4-hydroxylase (C4H), and 4-coumarate CoA ligase (4CL), providing precursors for the biosynthesis of all major phenolic secondary metabolites in higher organisms [34,35]. In this study, we noticed the activation of the phenylalanine metabolic pathway resulted in a significant upregulation of differential metabolites such as L-phenylalanine, L-tyrosine, and phenylacetic acid. Meanwhile, previous studies demonstrated that tyrosine and alanine are initiating molecules in the phenylalanine metabolic pathway, which can be converted to phenolic acid intermediates such as coumaric, ferulic, and butyric acids [36]. It can promote the accumulation of flavonoid phenolics, which may be one of the reasons for the differences in the antioxidant capacity of *F. luteovirens* in different regions. Therefore, a targeted metabolomic validation of flavonoid phenolics was performed we found the existence of differential metabolites vanillic acid, trans-cinnamic acid, salicylic acid, syringic acid, gentisic acid, 4-hydroxycinnamic acid, 2,6-dihydroxybenzoic acid, which are significantly different from each other.

Further, we found that the differences in the antioxidant capacity of *F. luteovirens* from different regions may be associated with environmental factors. Altitude plays a crucial role in shaping the environment, characterized by significant diurnal temperature fluctuations, reduced oxygen levels, frigid temperatures, and intense UV radiation [37,38]. Consequently, species inhabiting high altitudes necessitate the adoption of multiple strategies to effectively acclimate to harsh environmental conditions. Numerous previous studies suggested that environmental factors have a significant effect on the growth and development of organisms, especially on the accumulation of their flavonoid metabolites [39,40,41]. Environmental factors contribute to the flavonoid biosynthesis of many organisms and generate economic value. Metabolomic and transcriptomic analyses of *Cyclocarya paliurus* grown at different altitudes revealed that differences in flavonoid content and composition may also be associated with the upregulation of genes related to energy and protein synthesis [37], which is consistent with the conclusions reached in this study. Among the harsh environmental factors at high altitudes, exposure to high UV rays may be a major influence. UVB radiation at high altitudes was found to be a key environmental feature affecting the increase in total flavonoid content of Ginkgo biloba and suggested that the transcription factor *GbHY5* may play a key role in the response to UV-B radiation [39]. In our study, we found that there were fewer differential metabolites in QLFL and ZDFL with similar high altitudes, while there was a large difference with XHFL in low altitude, which indicated that altitude affects the growth of *F. luteovirens* and accumulation of flavonoids, which is the same conclusion as that reached by the previous study. In addition, some studies revealed a positive modulation of total flavonoids by altitude and mean temperature in studies on *Lycium barbarum* [42]. This suggests that although *F. luteovirens* are both grown on the Tibetan Plateau, environmental factors in different regions may be contributing factors to differences in quality. Furthermore, some studies demonstrated that phenylpropane biosynthesis is affected by environmental conditions [43], which is consistent with the findings of this study, where we found that key metabolites in the phenylpropanoid synthesis and metabolic pathway were upregulated to promote the tyrosine pathway and the TCA cycle to provide energy support.

Previous studies have found that phenols are the main antioxidants of many wild and cultivated mushrooms [44,45]. Flavonoids, a class of phenolic compounds, serve as both metabolites produced by organisms in response to abiotic stresses and active components that confer health benefits [46]. Reportedly, flavonoids exhibit a wide variety of pharmacological activities to prevent and control diseases of the cardiovascular and cerebrovascular systems, as well as respiratory diseases and have pharmacological effects, such as anti-inflammatory and antibacterial, hypoglycemic, ameliorate cognitive impairment, and antioxidant properties [35,47]. We found that *F. luteovirens* from different regions were rich in 34 flavonoid phenolics, including ellagic acid, trans-cinnamic acid, vanillic acid, and other active ingredients with anti-obesity and anti-aging properties [48,49]. Moreover, prior research has demonstrated that para-hydroxybenzoate catechins, gallic acid, and caffeic acid are the primary phenolic components present in extracts of *Pleurotus eryngii* and *Auricularia auricula-judae*, which exhibit potent antioxidant activities, and all extracts were found to have protective effects on H_2_O_2_-induced oxidative cell damage without toxicity [50]. The studies conducted on *Ganoderma lucidum*, *Flammulina velutipes*, and other fungi have consistently demonstrated a strong correlation between metabolites such as Gallic acid (GAE) and trans-Cinnamic acid [51]. Our findings align with these results. These findings are crucial for comprehending the antioxidant activity of *F. luteovirens* across diverse regions, offering valuable insights and potential applications in domains such as food or pharmaceutical development. Given the substantial evidence demonstrating that fungal species, habitats, life history stages, processing methods, extraction techniques [52,53,54], and other factors significantly influence the chemical composition and antioxidant capacity of fungi, it becomes imperative to employ a diverse range of technologies for harnessing fungi’s potential in enhancing human health and overall quality of life [1]. In conclusion, *F. luteovirens* from different regions had strong antioxidant capacity, but there was a difference in total antioxidant capacity between *F. luteovirens* from the other two various regions and XHFL, and the total antioxidant capacity was found to be correlated with flavonoid content. The differences were found to be mainly related to the synthesis and metabolism of phenylalanine by metabolomics, which affected the flavonoid content and caused the differences in antioxidant capacity.

## 5. Conclusions

To facilitate the comprehensive utilization of *F. luteovirens*, this study investigates the antioxidant activity and metabolite diversity of *F. luteovirens* from three distinct regions. Metabolites in *F. luteovirens* exhibited significant variation across different geographic regions, predominantly comprising lipids and lipid-like molecules as well as organic acids and derivatives. We identified the key metabolites and pathways potentially associated with divergent antioxidant activity. KEGG pathway indicated that phenylalanine, tyrosine, tryptophan biosynthesis, and phenylalanine metabolism may differ during the development of *F. luteovirens* in different regions. Antioxidant activity of *F. luteovirens* increased with increasing altitude. Therefore, the biological properties of *F. luteovirens* may primarily be attributed to their lipid, amino acid, and derivative content, as well as flavonoids and phenolics. These findings offer valuable insights into the antioxidant capacity and metabolic profile of *F. luteovirens*. However, metabolomics alone is insufficient for comprehensive studies. Therefore, targeted quantitative investigations on *F. luteovirens* metabolites should be considered a prerequisite for scientific advancement and effective utilization of its application potential. Furthermore, future research endeavors should focus on elucidating the correlation between metabolites and multiple pharmacological activities rather than solely relying on in vitro antioxidant activity assessments.

## Data Availability

Data will be made available upon request.

## Supplementary Materials

The following supporting information can be downloaded at https://www.mdpi.com/article/10.3390/antiox13050620/s1, Figure S1: Classification of differential metabolites; Table S1: Calibration curve; Table S2: BPC diagram; Table S3: Different metabolites; Table S4: Quantified 34 flavonoid-phenolic metabolites in *F. luteovirens.*
