# Supplementary material for: Metabolomic Profiling of Floccularia luteovirens from Different Geographical Regions Proposes a Novel Perspective on Their Antioxidative Activities
Source: Antioxidants (Basel). 2024 May 20;13(5):620. doi: 10.3390/antiox13050620 (PMC11118160; doi:10.3390/antiox13050620)

Calibration Curve

1

Analyte Name: (+/-)Catechin

Internal Standard: N/A

**Data File**	81089-BLK.wiff	**Result Table**	DZLM2023080667-results-20230907-5500(mix129)
**Acquisition Date**	8/21/2023 1:07:38 PM	**Algorithm Used**	MQ4
**Acquisition Method**	20230306-flavones(mix130-T3)-15min.dam	**Instrument Name**	Triple Quad 5500+
**Project**	N/A	**Processing Method**	20230329-flavones-(mix130-T3)-15min.qmethod

Regression Equation: y = 1025.06539 x + 866.27839 (r = 0.99897, r^2 = 0.99794)

(weighting: 1 / x)

Expected Concentration	Number of Values	Mean Calculated Concentration (ng/mL)	% Accuracy	Std. Deviation	%CV
0.02	0 of 1	N/A	N/A	N/A	N/A
0.05	0 of 1	N/A	N/A	N/A	N/A
0.13	0 of 1	N/A	N/A	N/A	N/A
0.33	0 of 1	N/A	N/A	N/A	N/A
0.82	0 of 1	N/A	N/A	N/A	N/A
2.05	0 of 1	N/A	N/A	N/A	N/A
5.12	1 of 1	5.960	116.4	N/A	N/A
12.80	1 of 1	11.008	86.0	N/A	N/A
32.00	1 of 1	31.529	98.5	N/A	N/A
80.00	1 of 1	77.810	97.3	N/A	N/A
200.00	1 of 1	203.613	101.8	N/A	N/A

Analyte Name: (+/-)Catechin

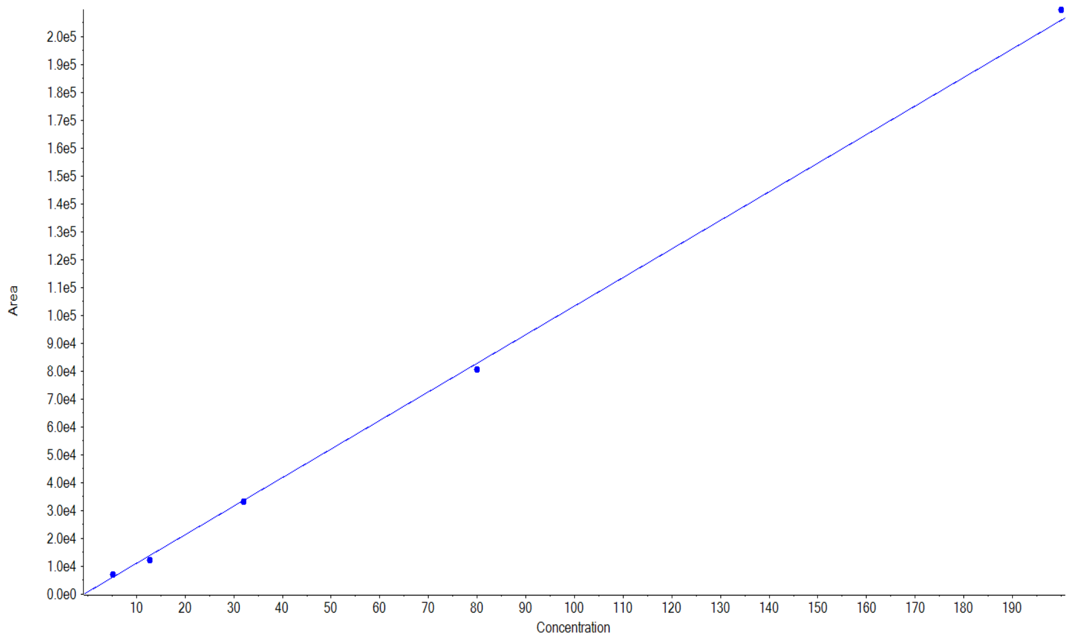

Analyte Name: 2,4-Dihydroxybenzoic acid
Internal Standard: N/A

**Data File**	81089-BLK.wiff	**Result Table**	DZLM2023080667-results-20230907-5500(mix129)
**Acquisition Date**	8/21/2023 1:07:38 PM	**Algorithm Used**	MQ4
**Acquisition Method**	20230306-flavones(mix130-T3)-15min.dam	**Instrument Name**	Triple Quad 5500+
**Project**	N/A	**Processing Method**	20230329-flavones-(mix130-T3)-15min.qmethod

Regression Equation: y = 2912.05208 x + 221.80873 (r = 0.99871, r² = 0.99742)
(weighting: 1 / x)

Expected Concentration	Number of Values	Mean Calculated Concentration (ng/mL)	% Accuracy	Std. Deviation	%CV
0.02	0 of 1	N/A	N/A	N/A	N/A
0.05	0 of 1	N/A	N/A	N/A	N/A
0.13	0 of 1	N/A	N/A	N/A	N/A
0.33	0 of 1	N/A	N/A	N/A	N/A
0.82	0 of 1	N/A	N/A	N/A	N/A
2.05	0 of 1	N/A	N/A	N/A	N/A
5.12	1 of 1	4.484	87.6	N/A	N/A
12.80	1 of 1	13.662	106.7	N/A	N/A
32.00	1 of 1	35.288	110.3	N/A	N/A
80.00	1 of 1	76.237	95.3	N/A	N/A
200.00	1 of 1	200.249	100.1	N/A	N/A

Analyte Name: 2,4-Dihydroxybenzoic acid

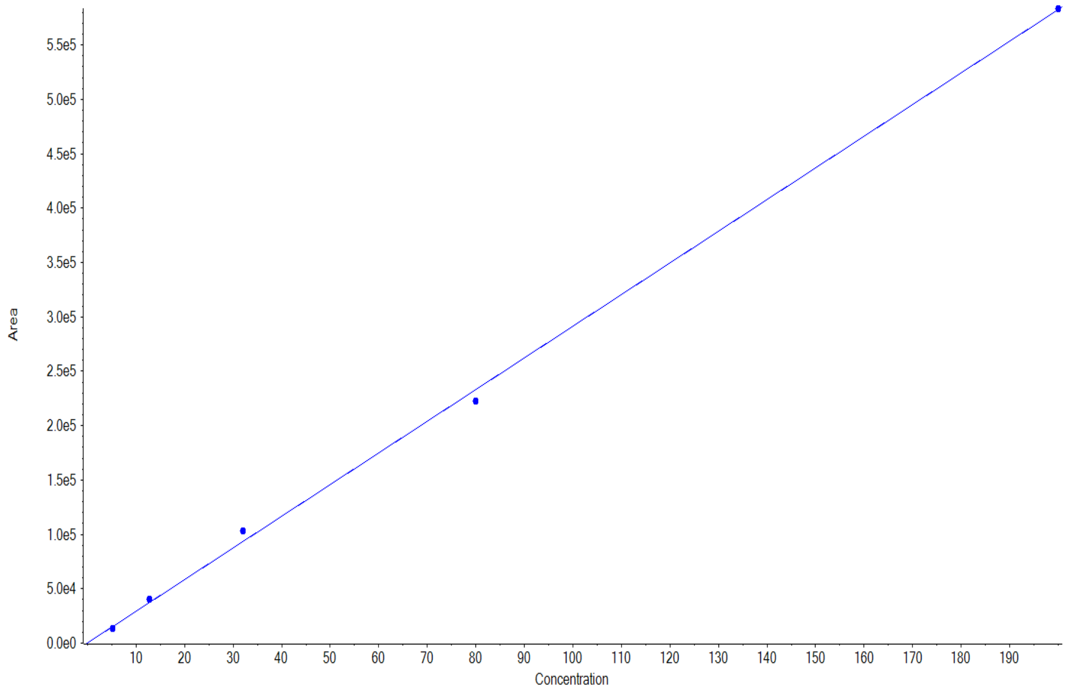

3

Analyte Name: 2,6-Dihydroxybenzoic acid

Internal Standard: N/A

**Data File**	81089-BLK.wiff	**Result Table**	DZLM2023080667-results-20230907-5500(mix129)
**Acquisition Date**	8/21/2023 1:07:38 PM	**Algorithm Used**	MQ4
**Acquisition Method**	20230306-flavones(mix130-T3)-15min.dam	**Instrument Name**	Triple Quad 5500+
**Project**	N/A	**Processing Method**	20230329-flavones-(mix130-T3)-15min.qmethod

Regression Equation: y = 14422.48851 x + -979.04142 (r = 0.99952, r^2 = 0.99904)

(weighting: 1 / x)

Expected Concentration	Number of Values	Mean Calculated Concentration (ng/mL)	% Accuracy	Std. Deviation	%CV
0.02	0 of 1	N/A	N/A	N/A	N/A
0.05	0 of 1	N/A	N/A	N/A	N/A
0.13	0 of 1	N/A	N/A	N/A	N/A
0.33	0 of 1	N/A	N/A	N/A	N/A
0.82	0 of 1	N/A	N/A	N/A	N/A
2.05	1 of 1	2.320	113.2	N/A	N/A
5.12	1 of 1	4.823	94.2	N/A	N/A
12.80	1 of 1	11.907	93.0	N/A	N/A
32.00	1 of 1	30.813	96.3	N/A	N/A
80.00	1 of 1	83.009	103.8	N/A	N/A
200.00	1 of 1	199.099	99.6	N/A	N/A

Analyte Name: 2,6-Dihydroxybenzoic acid

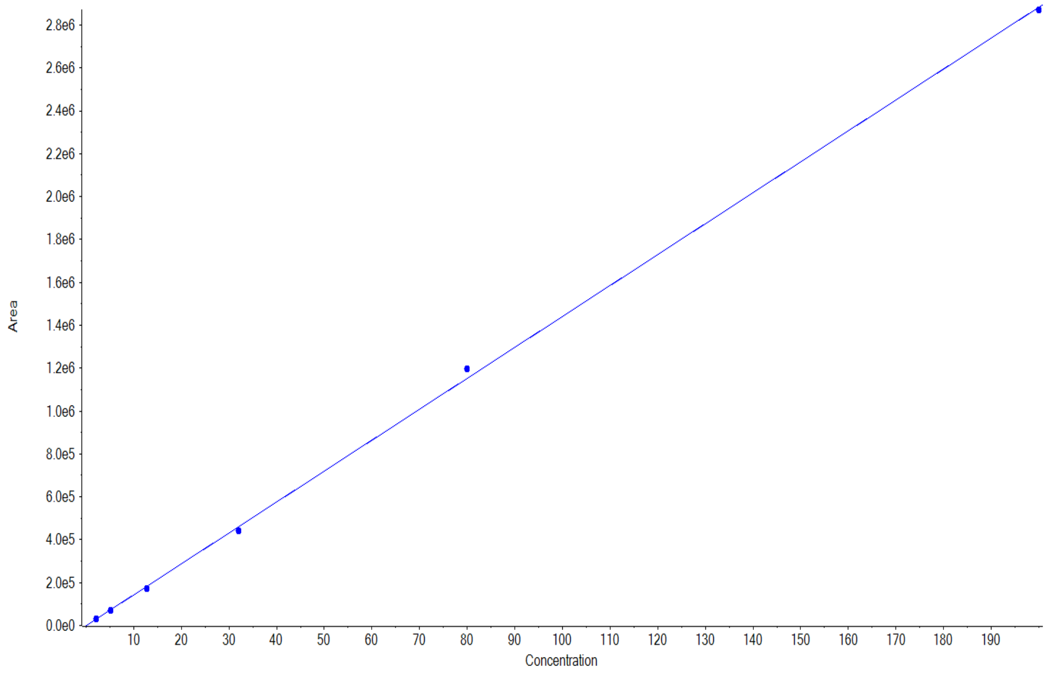

Analyte Name: 3-Hydroxyflavone

Internal Standard: N/A

**Data File**	81089-BLK.wiff	**Result Table**	DZLM2023080667-results-20230907-5500(mix129)
**Acquisition Date**	8/21/2023 1:07:38 PM	**Algorithm Used**	MQ4
**Acquisition Method**	20230306-flavones(mix130-T3)-15min.dam	**Instrument Name**	Triple Quad 5500+
**Project**	N/A	**Processing Method**	20230329-flavones-(mix130-T3)-15min.qmethod

Regression Equation: y = 25331.35672 x + -97.42076 (r = 0.99961, r² = 0.99923)

(weighting: 1 / x)

Expected Concentration	Number of Values	Mean Calculated Concentration (ng/mL)	% Accuracy	Std. Deviation	%CV
0.02	0 of 1	N/A	N/A	N/A	N/A
0.05	0 of 1	N/A	N/A	N/A	N/A
0.13	0 of 1	N/A	N/A	N/A	N/A
0.33	1 of 1	0.277	84.0	N/A	N/A
0.82	1 of 1	0.871	106.2	N/A	N/A
2.05	1 of 1	2.264	110.4	N/A	N/A
5.12	1 of 1	5.150	100.6	N/A	N/A
12.80	1 of 1	12.703	99.2	N/A	N/A
32.00	1 of 1	31.855	99.6	N/A	N/A
80.00	0 of 1	N/A	N/A	N/A	N/A
200.00	0 of 1	N/A	N/A	N/A	N/A

Analyte Name: 3-Hydroxyflavone

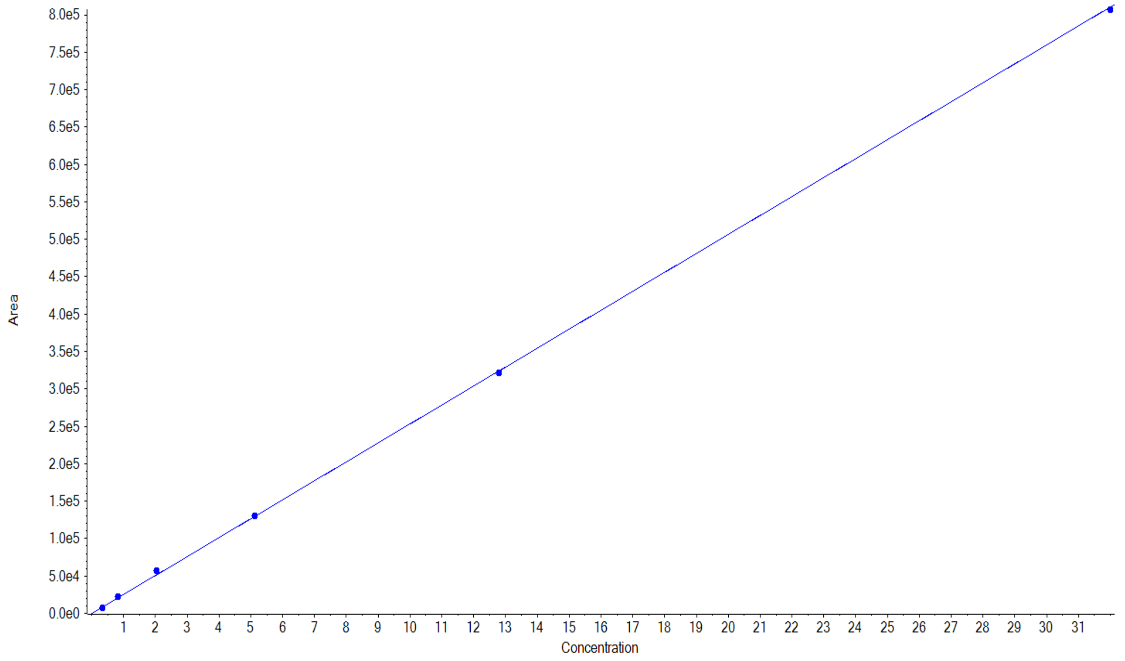

5

Analyte Name: 3,4-Dihydro-2H-1-benzopyran-2-one

Internal Standard: N/A

**Data File**	81089-BLK.wiff	**Result Table**	DZLM2023080667-results-20230907-5500(mix129)
**Acquisition Date**	8/21/2023 1:07:38 PM	**Algorithm Used**	MQ4
**Acquisition Method**	20230306-flavones(mix130-T3)-15min.dam	**Instrument Name**	Triple Quad 5500+
**Project**	N/A	**Processing Method**	20230329-flavones-(mix130-T3)-15min.qmethod

Regression Equation: y = 2354.53635 x + 534.34288 (r = 0.99973, r^2 = 0.99947)

(weighting: 1 / x)

Expected Concentration	Number of Values	Mean Calculated Concentration (ng/mL)	% Accuracy	Std. Deviation	%CV
0.02	0 of 1	N/A	N/A	N/A	N/A
0.05	0 of 1	N/A	N/A	N/A	N/A
0.13	0 of 1	N/A	N/A	N/A	N/A
0.33	0 of 1	N/A	N/A	N/A	N/A
0.82	0 of 1	N/A	N/A	N/A	N/A
2.05	1 of 1	1.871	91.3	N/A	N/A
5.12	1 of 1	5.124	100.1	N/A	N/A
12.80	1 of 1	13.347	104.3	N/A	N/A
32.00	1 of 1	33.639	105.1	N/A	N/A
80.00	1 of 1	80.354	100.4	N/A	N/A
200.00	1 of 1	197.634	98.8	N/A	N/A

Analyte Name: 3,4-Dihydro-2H-1-benzopyran-2-one

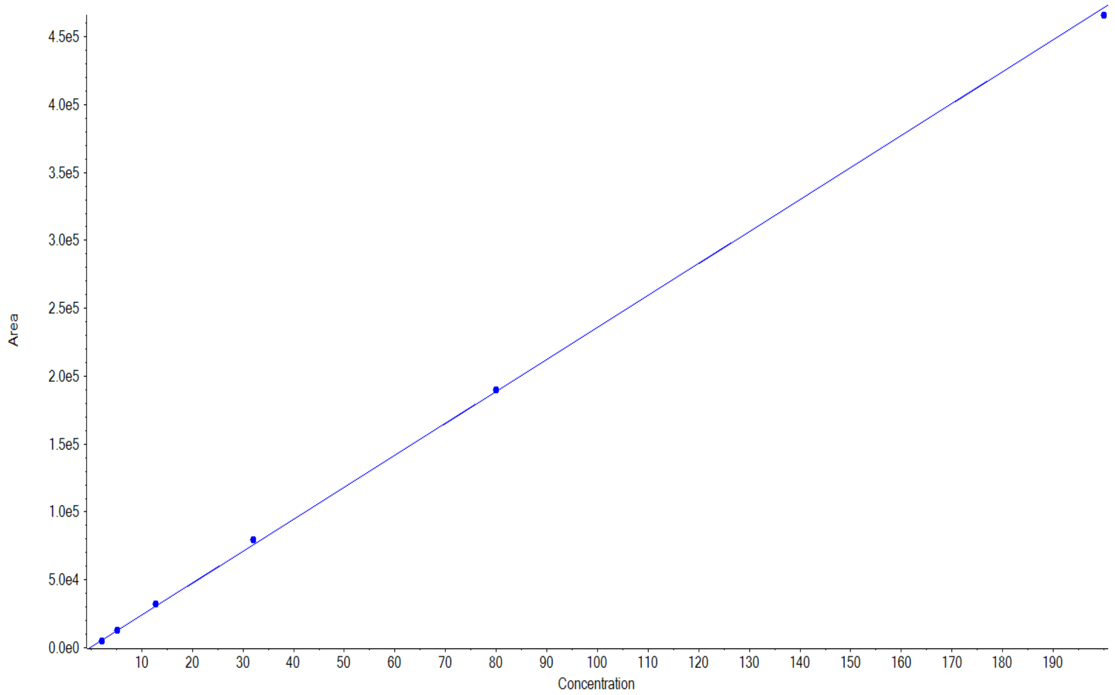

6

Analyte Name: 4-Hydroxybenzoic acid

Internal Standard: N/A

**Data File**	81089-BLK.wiff	**Result Table**	DZLM2023080667-results-20230907-5500(mix129)
**Acquisition Date**	8/21/2023 1:07:38 PM	**Algorithm Used**	MQ4
**Acquisition Method**	20230306-flavones(mix130-T3)-15min.dam	**Instrument Name**	Triple Quad 5500+
**Project**	N/A	**Processing Method**	20230329-flavones-(mix130-T3)-15min.qmethod

Regression Equation: y = 5.45589e4 x + 6.26826e4 (r = 0.99988, r^2 = 0.99975)

(weighting: 1 / x)

Expected Concentration	Number of Values	Mean Calculated Concentration (ng/mL)	% Accuracy	Std. Deviation	%CV
0.02	0 of 1	N/A	N/A	N/A	N/A
0.05	0 of 1	N/A	N/A	N/A	N/A
0.13	0 of 1	N/A	N/A	N/A	N/A
0.33	1 of 1	0.369	111.8	N/A	N/A
0.82	1 of 1	0.736	89.8	N/A	N/A
2.05	1 of 1	1.949	95.1	N/A	N/A
5.12	1 of 1	5.015	97.9	N/A	N/A
12.80	1 of 1	13.634	106.5	N/A	N/A
32.00	1 of 1	31.834	99.5	N/A	N/A
80.00	1 of 1	79.447	99.3	N/A	N/A
200.00	1 of 1	200.136	100.1	N/A	N/A

Analyte Name: 4-Hydroxybenzoic acid

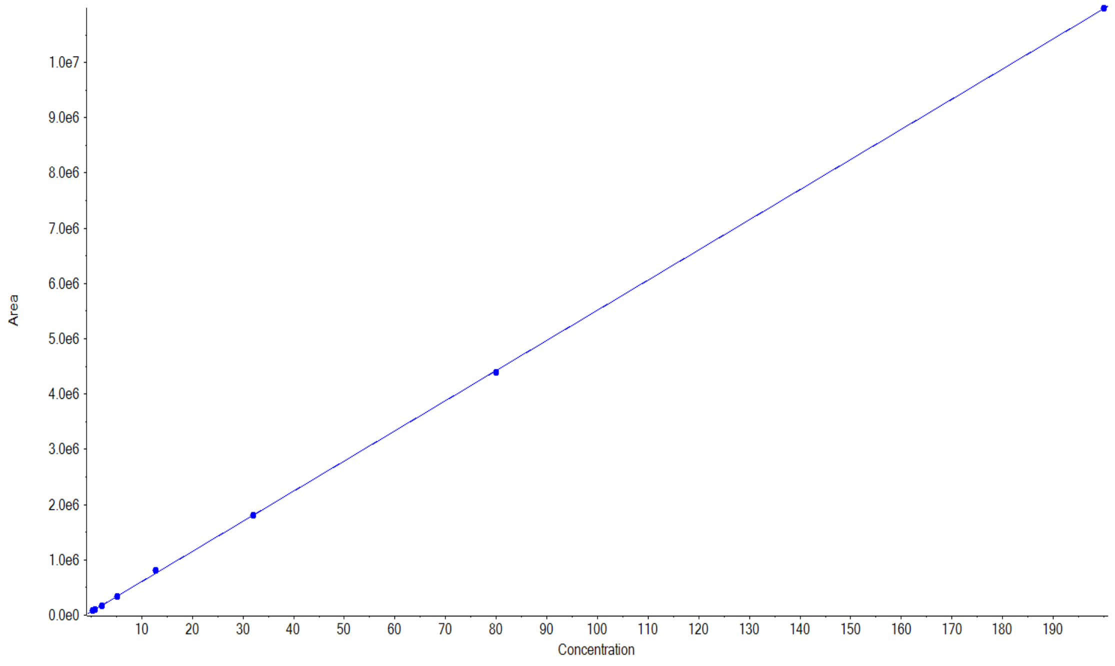

Analyte Name: 4-Hydroxycinnamic acid

Internal Standard: N/A

**Data File**	81089-BLK.wiff	**Result Table**	DZLM2023080667-results-20230907-5500(mix129)
**Acquisition Date**	8/21/2023 1:07:38 PM	**Algorithm Used**	MQ4
**Acquisition Method**	20230306-flavones(mix130-T3)-15min.dam	**Instrument Name**	Triple Quad 5500+
**Project**	N/A	**Processing Method**	20230329-flavones-(mix130-T3)-15min.qmethod

Regression Equation: y = 6.72884e4 x + 2202.87882 (r = 0.99979, r^2 = 0.99957)

(weighting: 1 / x)

Expected Concentration	Number of Values	Mean Calculated Concentration (ng/mL)	% Accuracy	Std. Deviation	%CV
0.02	0 of 1	N/A	N/A	N/A	N/A
0.05	0 of 1	N/A	N/A	N/A	N/A
0.13	0 of 1	N/A	N/A	N/A	N/A
0.33	0 of 1	N/A	N/A	N/A	N/A
0.82	1 of 1	0.960	117.1	N/A	N/A
2.05	1 of 1	1.972	96.2	N/A	N/A
5.12	1 of 1	4.756	92.9	N/A	N/A
12.80	1 of 1	12.138	94.8	N/A	N/A
32.00	1 of 1	31.097	97.2	N/A	N/A
80.00	1 of 1	81.188	101.5	N/A	N/A
200.00	1 of 1	200.678	100.3	N/A	N/A

Analyte Name: 4-Hydroxycinnamic acid

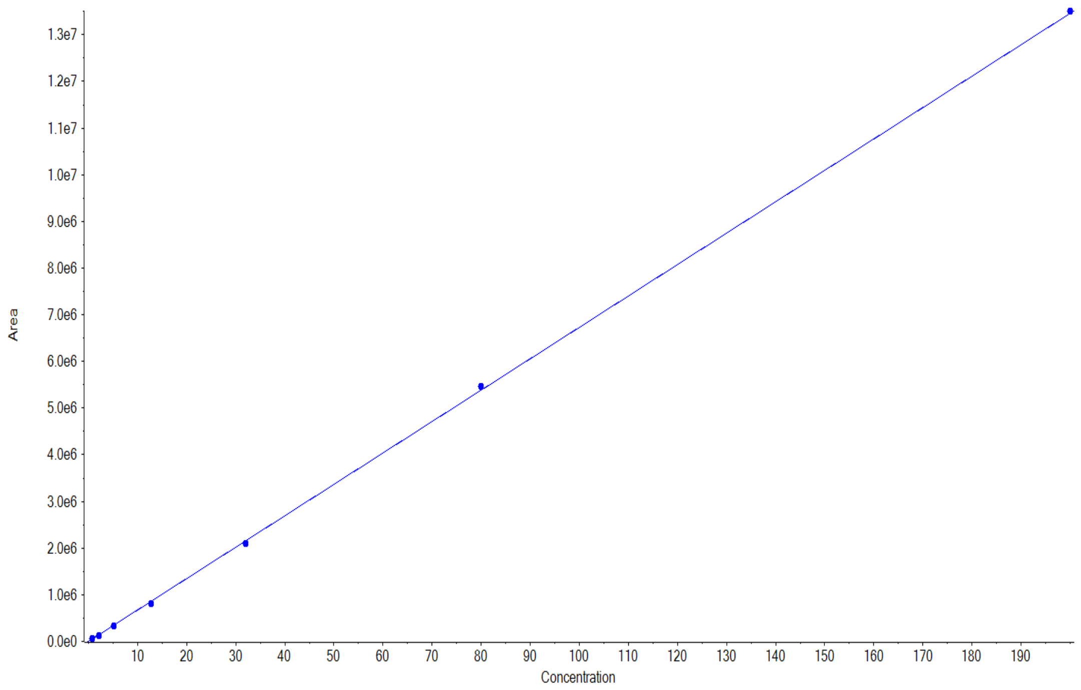

Analyte Name: 6-Methoxyflavone

Internal Standard: N/A

**Data File**	81089-BLK.wiff	**Result Table**	DZLM2023080667-results-20230907-5500(mix129)
**Acquisition Date**	8/21/2023 1:07:38 PM	**Algorithm Used**	MQ4
**Acquisition Method**	20230306-flavones(mix130-T3)-15min.dam	**Instrument Name**	Triple Quad 5500+
**Project**	N/A	**Processing Method**	20230329-flavones-(mix130-T3)-15min.qmethod

Regression Equation: y = 1.04742e5 x + -4230.66366 (r = 0.99896, r^2 = 0.99792)

(weighting: 1 / x)

Expected Concentration	Number of Values	Mean Calculated Concentration (ng/mL)	% Accuracy	Std. Deviation	%CV
0.02	0 of 1	N/A	N/A	N/A	N/A
0.05	0 of 1	N/A	N/A	N/A	N/A
0.13	1 of 1	0.131	101.1	N/A	N/A
0.33	1 of 1	0.345	104.5	N/A	N/A
0.82	1 of 1	0.812	99.0	N/A	N/A
2.05	1 of 1	1.997	97.4	N/A	N/A
5.12	1 of 1	5.265	102.8	N/A	N/A
12.80	1 of 1	11.832	92.4	N/A	N/A
32.00	1 of 1	32.868	102.7	N/A	N/A
80.00	0 of 1	N/A	N/A	N/A	N/A
200.00	0 of 1	N/A	N/A	N/A	N/A

Analyte Name: 6-Methoxyflavone

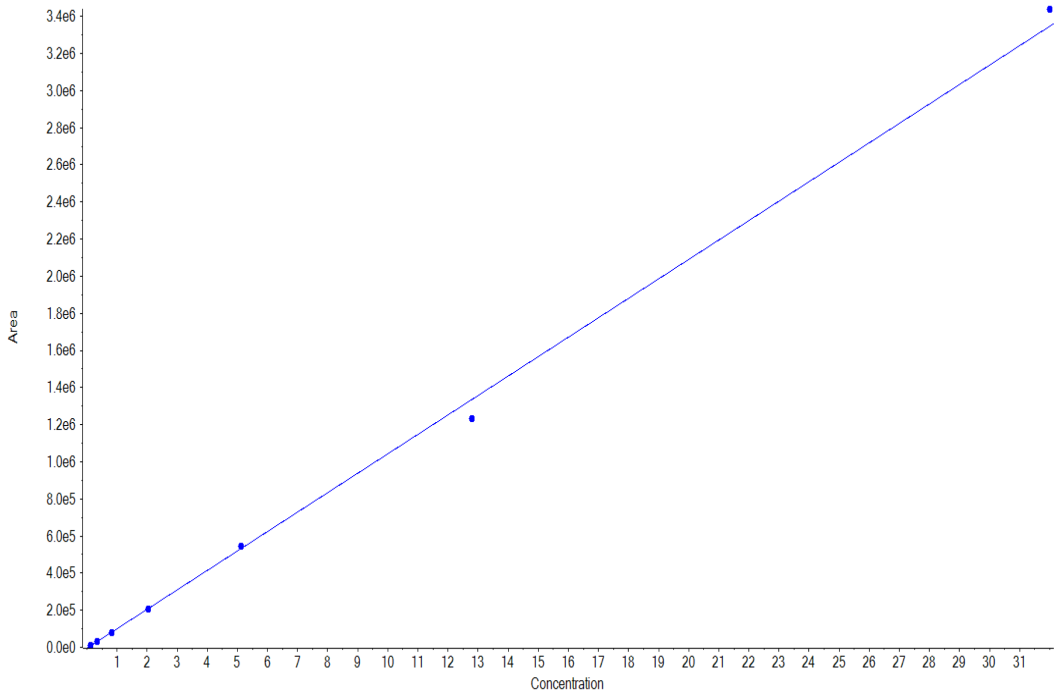

9

Analyte Name: Apigenin

Internal Standard: N/A

**Data File**	81089-BLK.wiff	**Result Table**	DZLM2023080667-results-20230907-5500(mix129)
**Acquisition Date**	8/21/2023 1:07:38 PM	**Algorithm Used**	MQ4
**Acquisition Method**	20230306-flavones(mix130-T3)-15min.dam	**Instrument Name**	Triple Quad 5500+
**Project**	N/A	**Processing Method**	20230329-flavones-(mix130-T3)-15min.qmethod

Regression Equation: y = 11749.02209 x + 184.73543 (r = 0.99956, r² = 0.99911)

(weighting: 1 / x)

Expected Concentration	Number of Values	Mean Calculated Concentration (ng/mL)	% Accuracy	Std. Deviation	%CV
0.02	1 of 1	0.041	206.7	N/A	N/A
0.05	0 of 1	N/A	N/A	N/A	N/A
0.13	1 of 1	0.051	39.5	N/A	N/A
0.33	1 of 1	0.269	81.6	N/A	N/A
0.82	1 of 1	0.630	76.9	N/A	N/A
2.05	1 of 1	1.928	94.1	N/A	N/A
5.12	1 of 1	5.241	102.4	N/A	N/A
12.80	1 of 1	12.086	94.4	N/A	N/A
32.00	1 of 1	32.860	102.7	N/A	N/A
80.00	1 of 1	82.389	103.0	N/A	N/A
200.00	1 of 1	197.775	98.9	N/A	N/A

Analyte Name: Apigenin

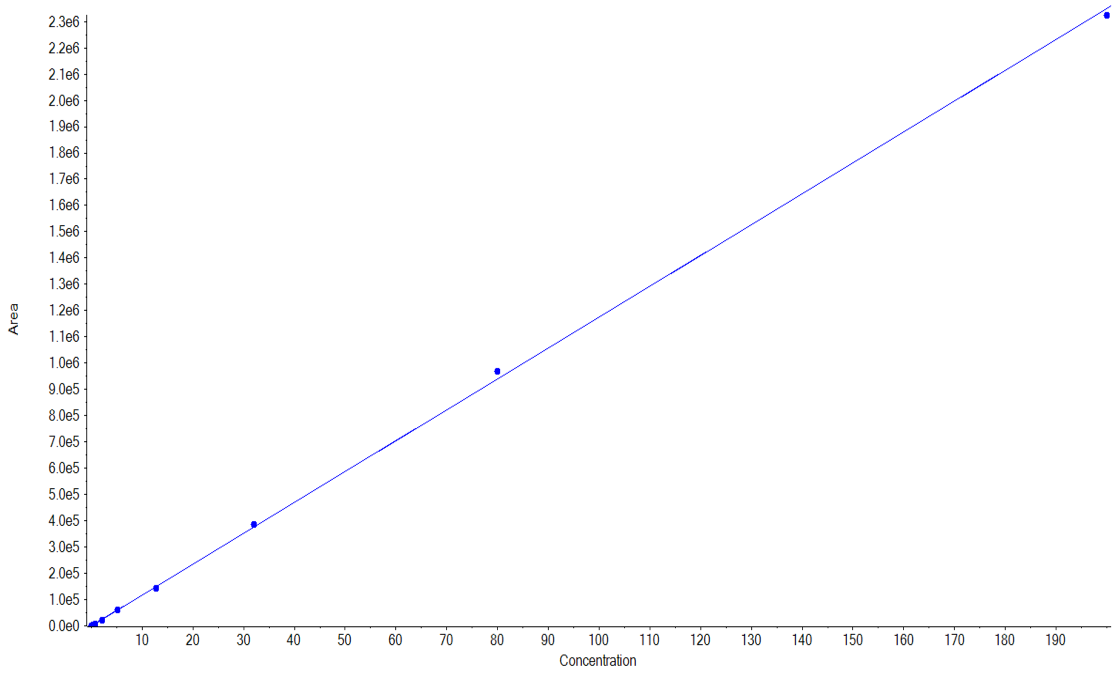

Analyte Name: Apiin

Internal Standard: N/A

**Data File**	81089-BLK.wiff	**Result Table**	DZLM2023080667-results-20230907-5500(mix129)
**Acquisition Date**	8/21/2023 1:07:38 PM	**Algorithm Used**	MQ4
**Acquisition Method**	20230306-flavones(mix130-T3)-15min.dam	**Instrument Name**	Triple Quad 5500+
**Project**	N/A	**Processing Method**	20230329-flavones-(mix130-T3)-15min.qmethod

Regression Equation: y = 5629.58496 x + -192.34363 (r = 0.99928, r^2 = 0.99857)

(weighting: 1 / x)

Expected Concentration	Number of Values	Mean Calculated Concentration (ng/mL)	% Accuracy	Std. Deviation	%CV
0.02	0 of 1	N/A	N/A	N/A	N/A
0.05	0 of 1	N/A	N/A	N/A	N/A
0.13	0 of 1	N/A	N/A	N/A	N/A
0.33	0 of 1	N/A	N/A	N/A	N/A
0.82	1 of 1	0.810	98.7	N/A	N/A
2.05	1 of 1	1.824	89.0	N/A	N/A
5.12	1 of 1	4.931	96.3	N/A	N/A
12.80	1 of 1	14.180	110.8	N/A	N/A
32.00	1 of 1	33.464	104.6	N/A	N/A
80.00	1 of 1	82.465	103.1	N/A	N/A
200.00	1 of 1	195.116	97.6	N/A	N/A

Analyte Name: Apiin

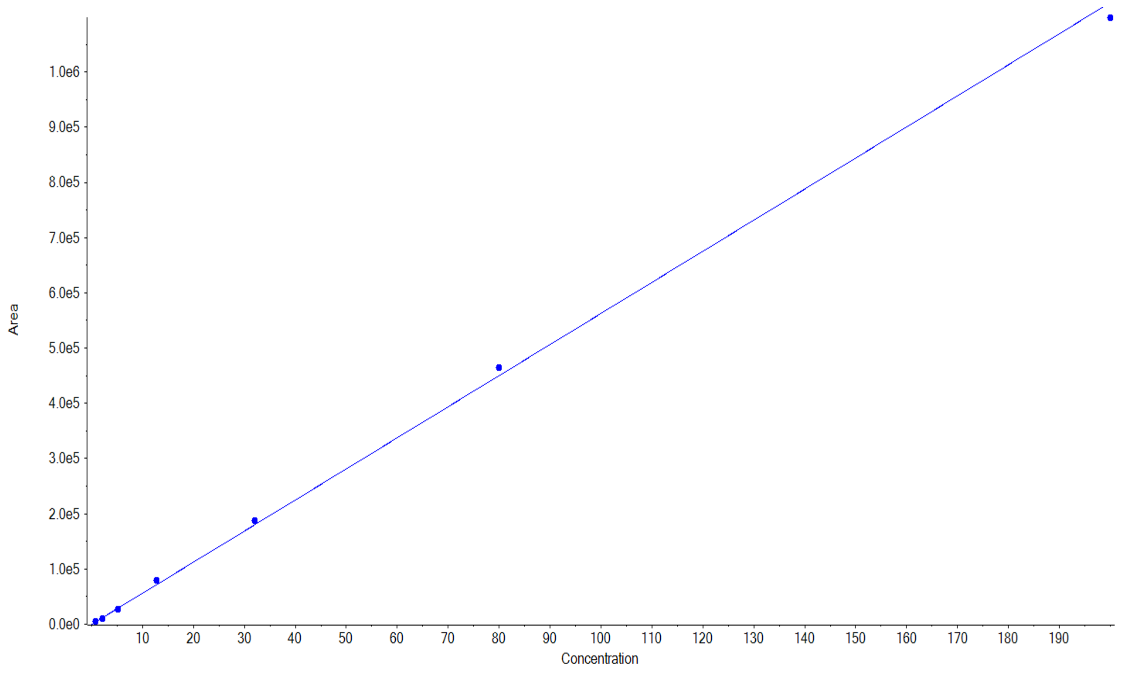

11

Analyte Name: Chlorogenic acid

Internal Standard: N/A

**Data File**	81089-BLK.wiff	**Result Table**	DZLM2023080667-results-20230907-5500(mix129)
**Acquisition Date**	8/21/2023 1:07:38 PM	**Algorithm Used**	MQ4
**Acquisition Method**	20230306-flavones(mix130-T3)-15min.dam	**Instrument Name**	Triple Quad 5500+
**Project**	N/A	**Processing Method**	20230329-flavones-(mix130-T3)-15min.qmethod

Regression Equation: y = 11855.82734 x + -1310.40422 (r = 0.99844, r² = 0.99688) (weighting: 1 / x)

Expected Concentration	Number of Values	Mean Calculated Concentration (ng/mL)	% Accuracy	Std. Deviation	%CV
0.02	0 of 1	N/A	N/A	N/A	N/A
0.05	0 of 1	N/A	N/A	N/A	N/A
0.13	0 of 1	N/A	N/A	N/A	N/A
0.33	1 of 1	0.297	90.0	N/A	N/A
0.82	1 of 1	0.761	92.8	N/A	N/A
2.05	1 of 1	2.107	102.8	N/A	N/A
5.12	1 of 1	5.640	110.2	N/A	N/A
12.80	1 of 1	12.816	100.1	N/A	N/A
32.00	1 of 1	34.572	108.0	N/A	N/A
80.00	1 of 1	76.927	96.2	N/A	N/A
200.00	0 of 1	N/A	N/A	N/A	N/A

Analyte Name: Chlorogenic acid

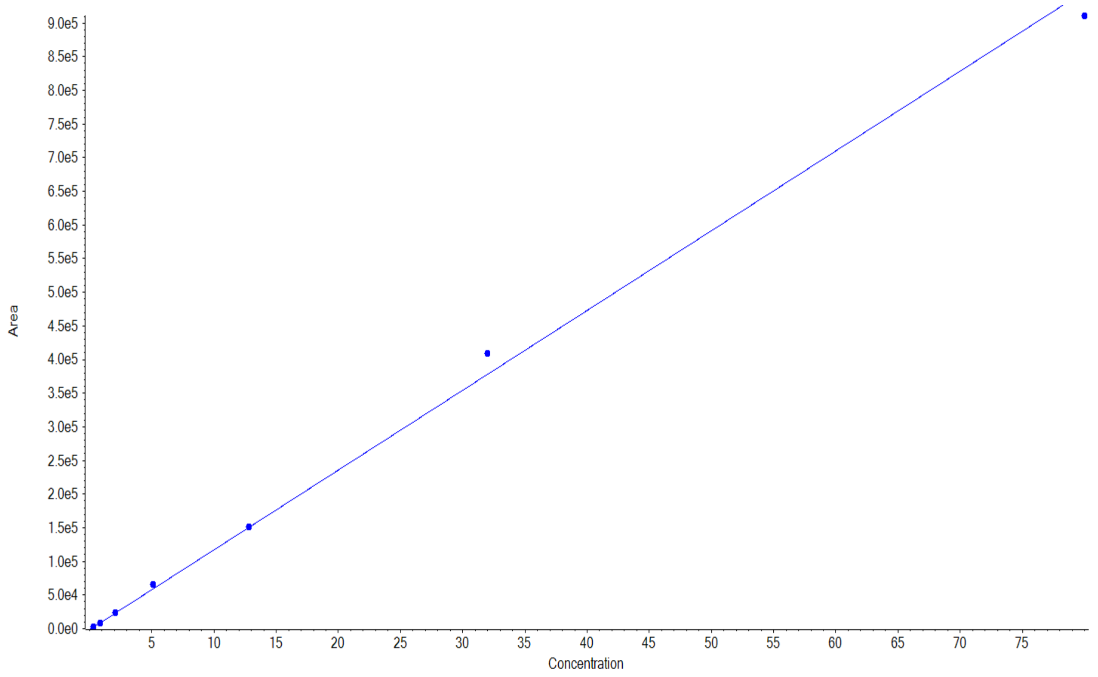

12

Analyte Name: LM_flavones_53

Internal Standard: N/A

**Data File**	81089-BLK.wiff	**Result Table**	DZLM2023080667-results-20230907-5500(mix129)
**Acquisition Date**	8/21/2023 1:07:38 PM	**Algorithm Used**	MQ4
**Acquisition Method**	20230306-flavones(mix130-T3)-15min.dam	**Instrument Name**	Triple Quad 5500+
**Project**	N/A	**Processing Method**	20230329-flavones-(mix130-T3)-15min.qmethod

Regression Equation: y = 13263.59821 x + -871.68156 (r = 0.99982, r^2 = 0.99964)

(weighting: 1 / x)

Expected Concentration	Number of Values	Mean Calculated Concentration (ng/mL)	% Accuracy	Std. Deviation	%CV
0.02	0 of 1	N/A	N/A	N/A	N/A
0.05	0 of 1	N/A	N/A	N/A	N/A
0.13	0 of 1	N/A	N/A	N/A	N/A
0.33	0 of 1	N/A	N/A	N/A	N/A
0.82	0 of 1	N/A	N/A	N/A	N/A
2.05	1 of 1	2.009	98.0	N/A	N/A
5.12	1 of 1	5.430	106.1	N/A	N/A
12.80	1 of 1	12.544	98.0	N/A	N/A
32.00	1 of 1	31.807	99.4	N/A	N/A
80.00	1 of 1	77.960	97.5	N/A	N/A
200.00	1 of 1	202.220	101.1	N/A	N/A

Analyte Name: LM_flavones_53

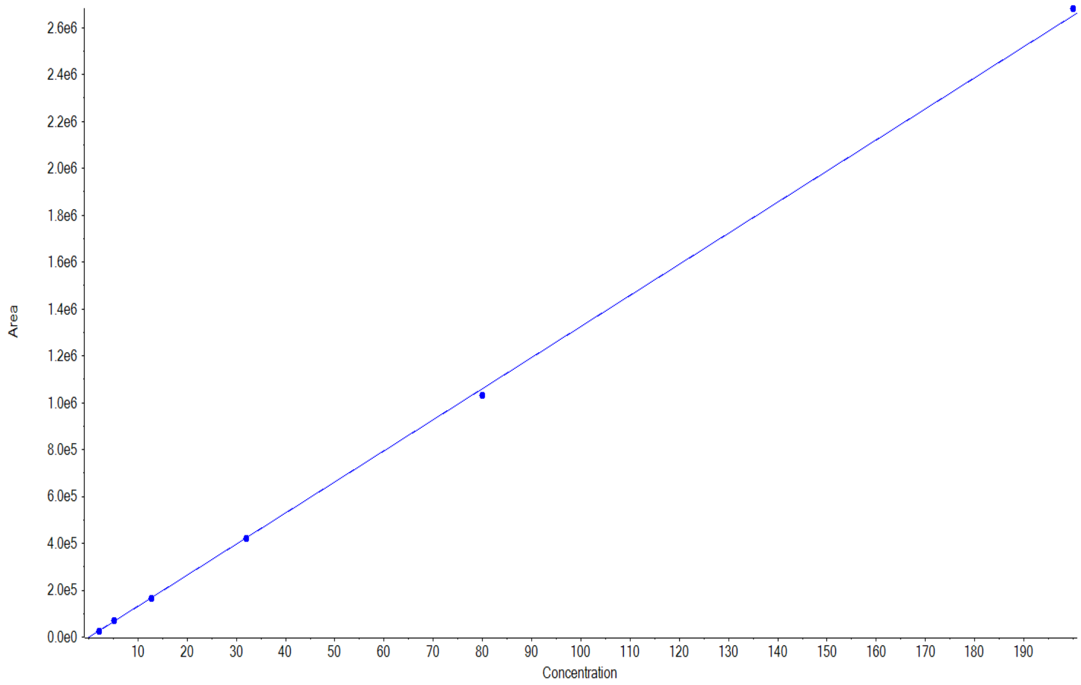

Analyte Name: Diosmin

Internal Standard: N/A

**Data File**	81089-BLK.wiff	**Result Table**	DZLM2023080667-results-20230907-5500(mix129)
**Acquisition Date**	8/21/2023 1:07:38 PM	**Algorithm Used**	MQ4
**Acquisition Method**	20230306-flavones(mix130-T3)-15min.dam	**Instrument Name**	Triple Quad 5500+
**Project**	N/A	**Processing Method**	20230329-flavones-(mix130-T3)-15min.qmethod

Regression Equation: y = 9052.83477 x + 4158.17746 (r = 0.99826, r^2 = 0.99652)

(weighting: 1 / x)

Expected Concentration	Number of Values	Mean Calculated Concentration (ng/mL)	% Accuracy	Std. Deviation	%CV
0.02	0 of 1	N/A	N/A	N/A	N/A
0.05	0 of 1	N/A	N/A	N/A	N/A
0.13	0 of 1	N/A	N/A	N/A	N/A
0.33	0 of 1	N/A	N/A	N/A	N/A
0.82	0 of 1	N/A	N/A	N/A	N/A
2.05	1 of 1	1.787	87.2	N/A	N/A
5.12	1 of 1	5.207	101.7	N/A	N/A
12.80	1 of 1	12.960	101.3	N/A	N/A
32.00	1 of 1	33.938	106.1	N/A	N/A
80.00	1 of 1	86.370	108.0	N/A	N/A
200.00	1 of 1	191.708	95.9	N/A	N/A

Analyte Name: Diosmin

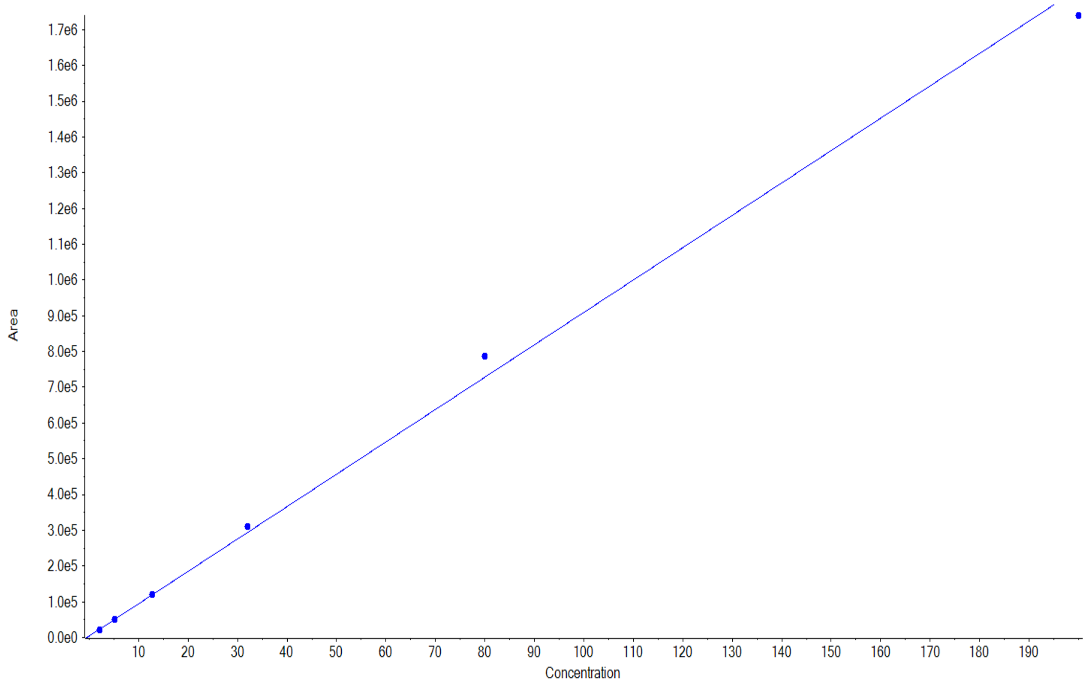

14

Analyte Name: Ellagic acid

Internal Standard: N/A

**Data File**	81089-BLK.wiff	**Result Table**	DZLM2023080667-results-20230907-5500(mix129)
**Acquisition Date**	8/21/2023 1:07:38 PM	**Algorithm Used**	MQ4
**Acquisition Method**	20230306-flavones(mix130-T3)-15min.dam	**Instrument Name**	Triple Quad 5500+
**Project**	N/A	**Processing Method**	20230329-flavones-(mix130-T3)-15min.qmethod

Regression Equation: y = 226.41777 x + -631.01074 (r = 0.99591, r² = 0.99183)

(weighting: 1 / x)

Expected Concentration	Number of Values	Mean Calculated Concentration (ng/mL)	% Accuracy	Std. Deviation	%CV
0.02	0 of 1	N/A	N/A	N/A	N/A
0.05	0 of 1	N/A	N/A	N/A	N/A
0.13	0 of 1	N/A	N/A	N/A	N/A
0.33	0 of 1	N/A	N/A	N/A	N/A
0.82	0 of 1	N/A	N/A	N/A	N/A
2.05	0 of 1	N/A	N/A	N/A	N/A
5.12	1 of 1	6.060	118.4	N/A	N/A
12.80	1 of 1	12.779	99.8	N/A	N/A
32.00	1 of 1	27.249	85.2	N/A	N/A
80.00	1 of 1	72.994	91.2	N/A	N/A
200.00	1 of 1	210.839	105.4	N/A	N/A

Analyte Name: Ellagic acid

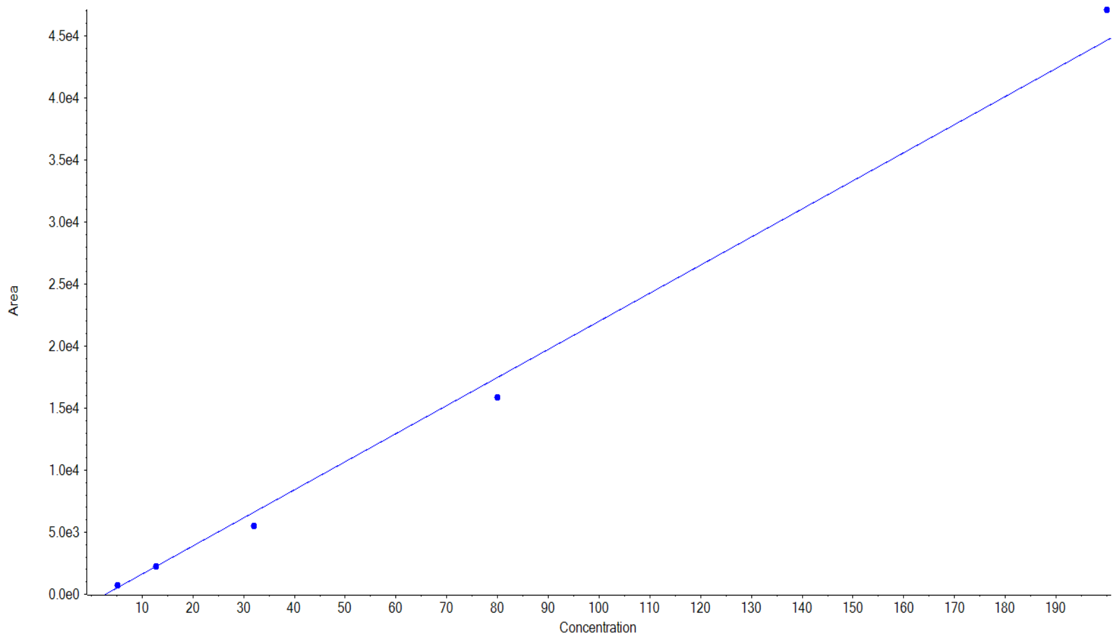

Analyte Name: Formononetin

Internal Standard: N/A

**Data File**	81089-BLK.wiff	**Result Table**	DZLM2023080667-results-20230907-5500(mix129)
**Acquisition Date**	8/21/2023 1:07:38 PM	**Algorithm Used**	MQ4
**Acquisition Method**	20230306-flavones(mix130-T3)-15min.dam	**Instrument Name**	Triple Quad 5500+
**Project**	N/A	**Processing Method**	20230329-flavones-(mix130-T3)-15min.qmethod

Regression Equation: y = 7.34645e4 x + -820.66920 (r = 0.99993, r² = 0.99985)

(weighting: 1 / x)

Expected Concentration	Number of Values	Mean Calculated Concentration (ng/mL)	% Accuracy	Std. Deviation	%CV
0.02	1 of 1	0.020	98.8	N/A	N/A
0.05	1 of 1	0.057	113.4	N/A	N/A
0.13	1 of 1	0.141	108.3	N/A	N/A
0.33	1 of 1	0.306	92.9	N/A	N/A
0.82	1 of 1	0.819	99.9	N/A	N/A
2.05	1 of 1	1.894	92.4	N/A	N/A
5.12	1 of 1	5.007	97.8	N/A	N/A
12.80	1 of 1	12.451	97.3	N/A	N/A
32.00	1 of 1	31.856	99.6	N/A	N/A
80.00	1 of 1	79.170	99.0	N/A	N/A
200.00	1 of 1	201.600	100.8	N/A	N/A

Analyte Name: Formononetin

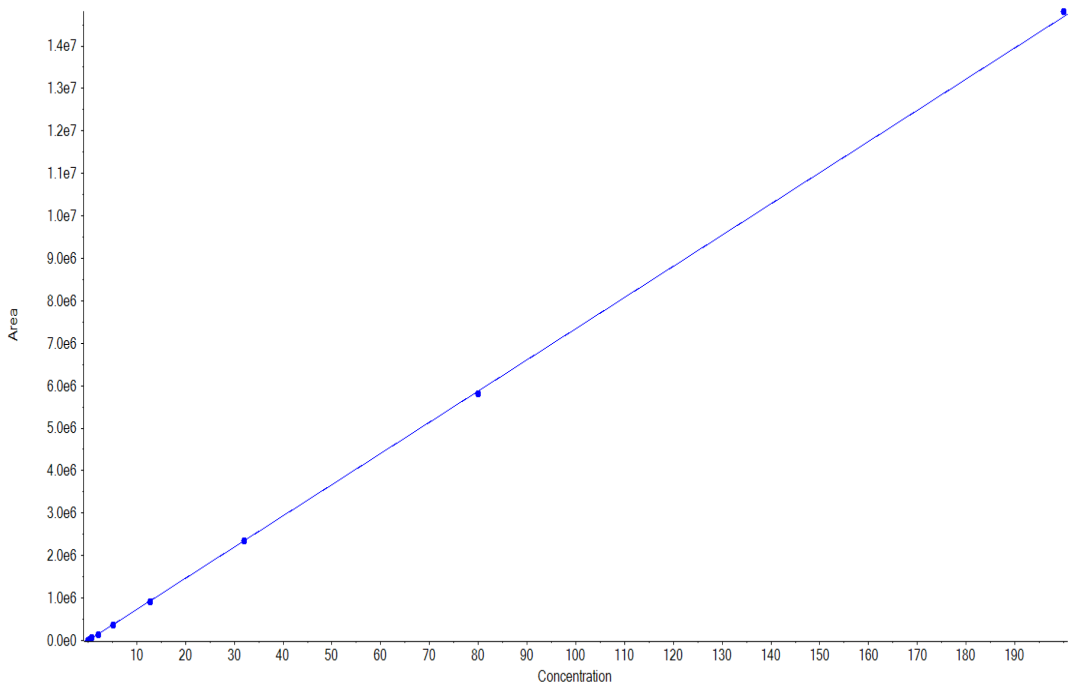

16

Analyte Name: Gallic acid

Internal Standard: N/A

**Data File**	81089-BLK.wiff	**Result Table**	DZLM2023080667-results-20230907-5500(mix129)
**Acquisition Date**	8/21/2023 1:07:38 PM	**Algorithm Used**	MQ4
**Acquisition Method**	20230306-flavones(mix130-T3)-15min.dam	**Instrument Name**	Triple Quad 5500+
**Project**	N/A	**Processing Method**	20230329-flavones-(mix130-T3)-15min.qmethod

Regression Equation: y = 10600.58342 x + 4684.22842 (r = 0.99893, r^2 = 0.99787)

(weighting: 1 / x)

Expected Concentration	Number of Values	Mean Calculated Concentration (ng/mL)	% Accuracy	Std. Deviation	%CV
0.02	0 of 1	N/A	N/A	N/A	N/A
0.05	0 of 1	N/A	N/A	N/A	N/A
0.13	0 of 1	N/A	N/A	N/A	N/A
0.33	0 of 1	N/A	N/A	N/A	N/A
0.82	1 of 1	0.702	85.6	N/A	N/A
2.05	1 of 1	2.168	105.8	N/A	N/A
5.12	1 of 1	5.570	108.8	N/A	N/A
12.80	1 of 1	13.081	102.2	N/A	N/A
32.00	1 of 1	31.270	97.7	N/A	N/A
80.00	0 of 1	N/A	N/A	N/A	N/A
200.00	0 of 1	N/A	N/A	N/A	N/A

Analyte Name: Gallic acid

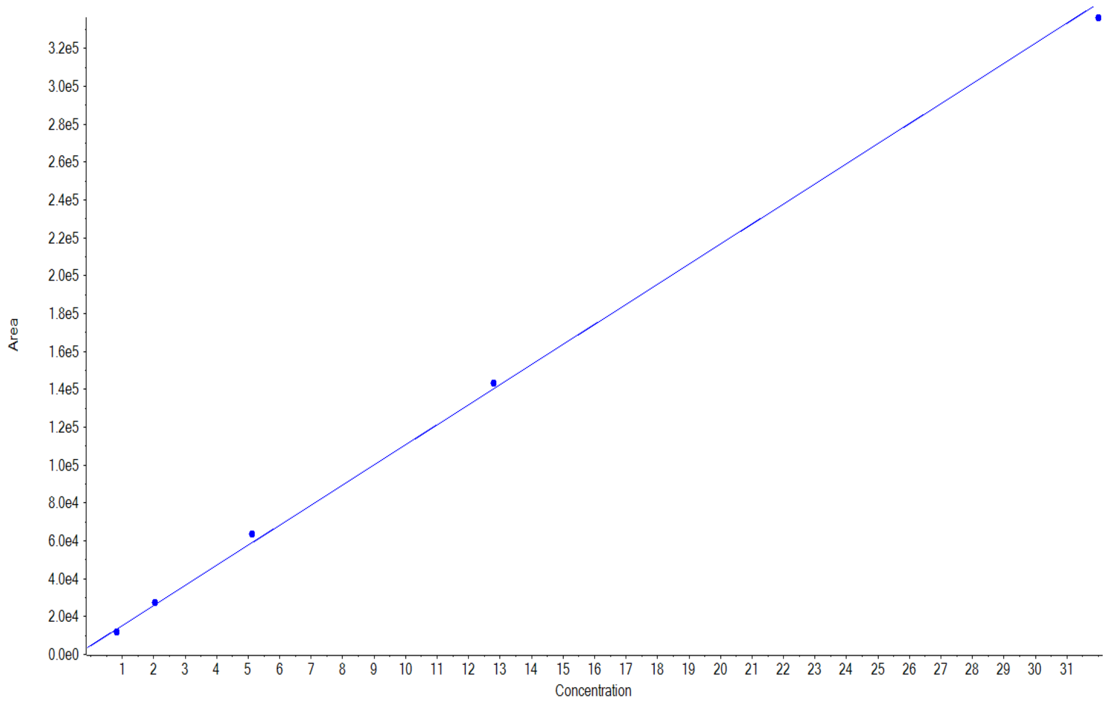

Analyte Name: Genistein

Internal Standard: N/A

**Data File**	81089-BLK.wiff	**Result Table**	DZLM2023080667-results-20230907-5500(mix129)
**Acquisition Date**	8/21/2023 1:07:38 PM	**Algorithm Used**	MQ4
**Acquisition Method**	20230306-flavones(mix130-T3)-15min.dam	**Instrument Name**	Triple Quad 5500+
**Project**	N/A	**Processing Method**	20230329-flavones-(mix130-T3)-15min.qmethod

Regression Equation: y = 3027.48083 x + -68.68956 (r = 0.99942, r^2 = 0.99884)

(weighting: 1 / x)

Expected Concentration	Number of Values	Mean Calculated Concentration (ng/mL)	% Accuracy	Std. Deviation	%CV
0.02	0 of 1	N/A	N/A	N/A	N/A
0.05	0 of 1	N/A	N/A	N/A	N/A
0.13	0 of 1	N/A	N/A	N/A	N/A
0.33	0 of 1	N/A	N/A	N/A	N/A
0.82	1 of 1	0.929	113.3	N/A	N/A
2.05	1 of 1	1.758	85.8	N/A	N/A
5.12	1 of 1	4.964	97.0	N/A	N/A
12.80	1 of 1	12.268	95.8	N/A	N/A
32.00	1 of 1	34.768	108.7	N/A	N/A
80.00	1 of 1	80.645	100.8	N/A	N/A
200.00	1 of 1	197.458	98.7	N/A	N/A

Analyte Name: Genistein

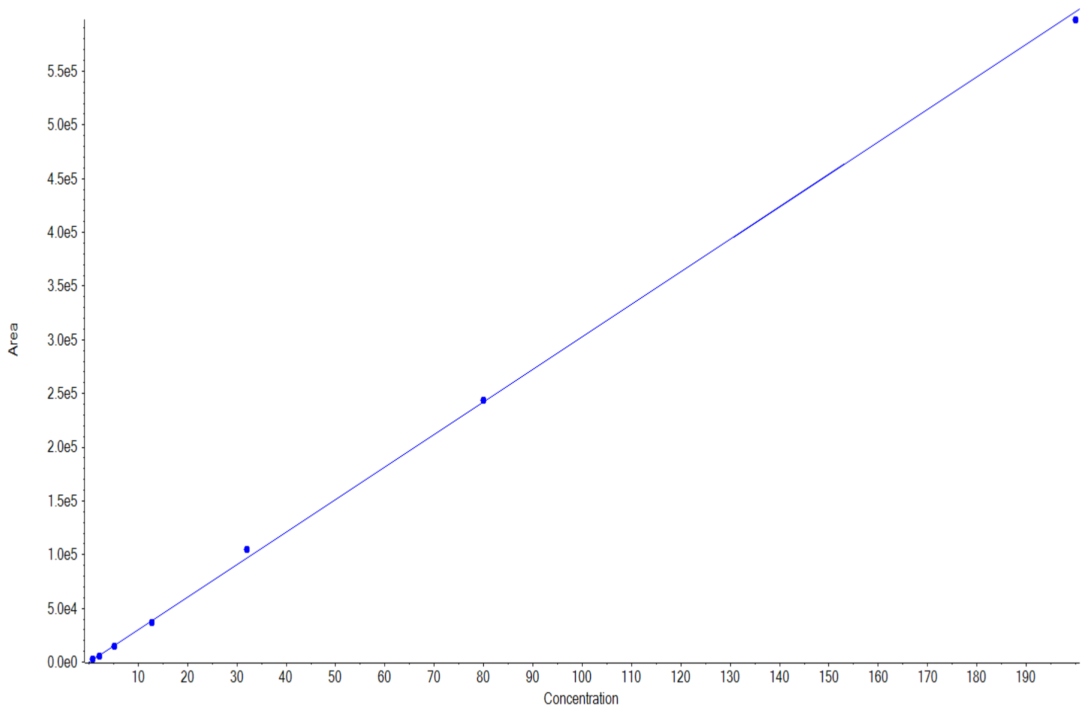

18

Analyte Name: Gentisic acid

Internal Standard: N/A

**Data File**	81089-BLK.wiff	**Result Table**	DZLM2023080667-results-20230907-5500(mix129)
**Acquisition Date**	8/21/2023 1:07:38 PM	**Algorithm Used**	MQ4
**Acquisition Method**	20230306-flavones(mix130-T3)-15min.dam	**Instrument Name**	Triple Quad 5500+
**Project**	N/A	**Processing Method**	20230329-flavones-(mix130-T3)-15min.qmethod

Regression Equation: y = 3103.89593 x + 4052.98663 (r = 0.99880, r^2 = 0.99760)

(weighting: 1 / x)

Expected Concentration	Number of Values	Mean Calculated Concentration (ng/mL)	% Accuracy	Std. Deviation	%CV
0.02	0 of 1	N/A	N/A	N/A	N/A
0.05	0 of 1	N/A	N/A	N/A	N/A
0.13	0 of 1	N/A	N/A	N/A	N/A
0.33	0 of 1	N/A	N/A	N/A	N/A
0.82	0 of 1	N/A	N/A	N/A	N/A
2.05	0 of 1	N/A	N/A	N/A	N/A
5.12	1 of 1	4.708	92.0	N/A	N/A
12.80	1 of 1	14.643	114.4	N/A	N/A
32.00	1 of 1	31.033	97.0	N/A	N/A
80.00	1 of 1	75.881	94.9	N/A	N/A
200.00	1 of 1	203.656	101.8	N/A	N/A

Analyte Name: Gentisic acid

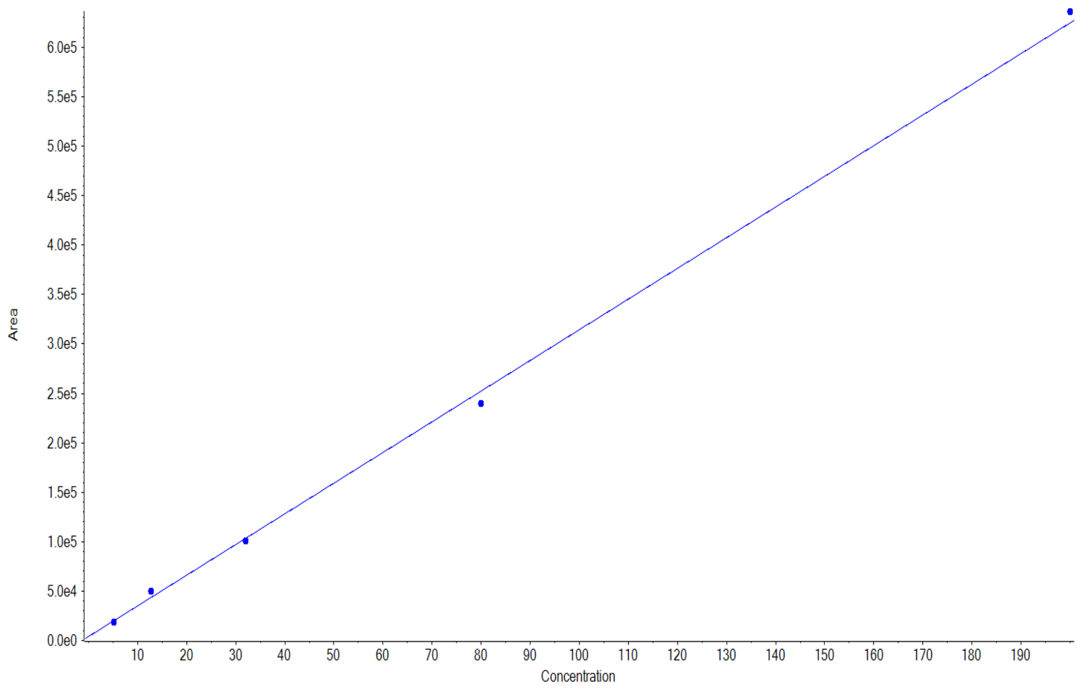

Analyte Name: Isorhamnetin

Internal Standard: N/A

**Data File**	81089-BLK.wiff	**Result Table**	DZLM2023080667-results-20230907-5500(mix129)
**Acquisition Date**	8/21/2023 1:07:38 PM	**Algorithm Used**	MQ4
**Acquisition Method**	20230306-flavones(mix130-T3)-15min.dam	**Instrument Name**	Triple Quad 5500+
**Project**	N/A	**Processing Method**	20230329-flavones-(mix130-T3)-15min.qmethod

Regression Equation: y = 25384.05898 x + -8627.86504 (r = 0.99930, r^2 = 0.99860) (weighting: 1 / x)

Expected Concentration	Number of Values	Mean Calculated Concentration (ng/mL)	% Accuracy	Std. Deviation	%CV
0.02	0 of 1	N/A	N/A	N/A	N/A
0.05	0 of 1	N/A	N/A	N/A	N/A
0.13	0 of 1	N/A	N/A	N/A	N/A
0.33	0 of 1	N/A	N/A	N/A	N/A
0.82	1 of 1	0.962	117.4	N/A	N/A
2.05	1 of 1	2.067	100.9	N/A	N/A
5.12	1 of 1	4.902	95.7	N/A	N/A
12.80	1 of 1	11.601	90.6	N/A	N/A
32.00	1 of 1	30.829	96.3	N/A	N/A
80.00	1 of 1	77.137	96.4	N/A	N/A
200.00	1 of 1	205.292	102.7	N/A	N/A

Analyte Name: Isorhamnetin

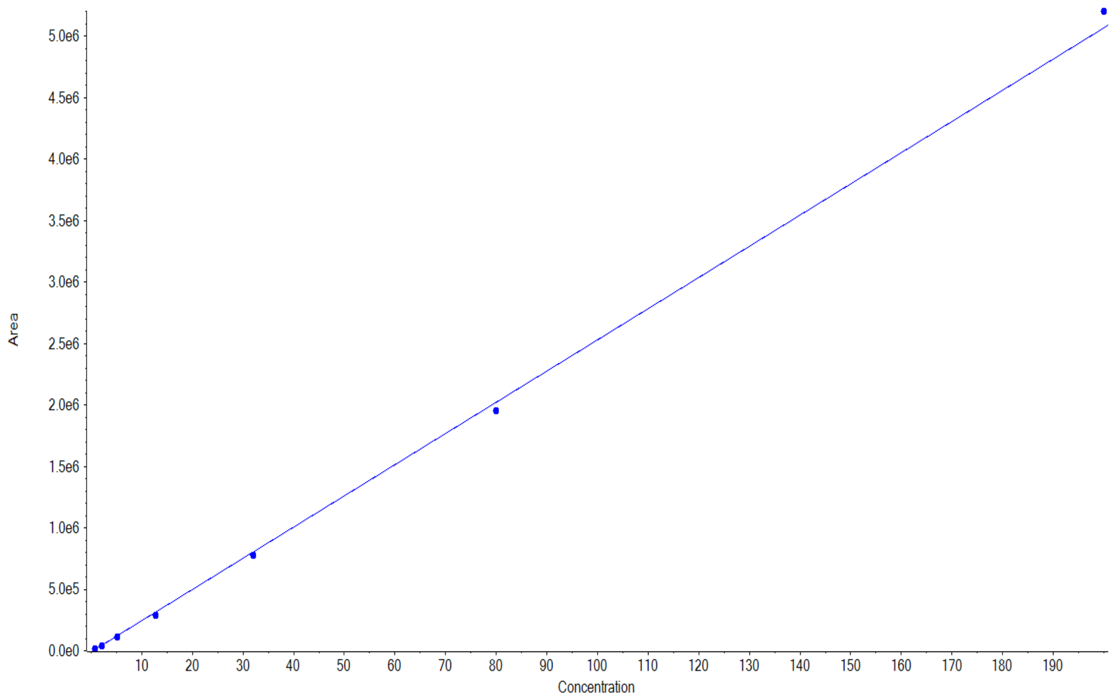

Analyte Name: Luteolin

Internal Standard: N/A

**Data File**	81089-BLK.wiff	**Result Table**	DZLM2023080667-results-20230907-5500(mix129)
**Acquisition Date**	8/21/2023 1:07:38 PM	**Algorithm Used**	MQ4
**Acquisition Method**	20230306-flavones(mix130-T3)-15min.dam	**Instrument Name**	Triple Quad 5500+
**Project**	N/A	**Processing Method**	20230329-flavones-(mix130-T3)-15min.qmethod

Regression Equation: y = 20535.22426 x + -122.54077 (r = 0.99953, r^2 = 0.99905)

(weighting: 1 / x)

Expected Concentration	Number of Values	Mean Calculated Concentration (ng/mL)	% Accuracy	Std. Deviation	%CV
0.02	1 of 1	0.028	138.9	N/A	N/A
0.05	1 of 1	0.051	101.5	N/A	N/A
0.13	0 of 1	N/A	N/A	N/A	N/A
0.33	0 of 1	N/A	N/A	N/A	N/A
0.82	1 of 1	0.736	89.7	N/A	N/A
2.05	1 of 1	1.802	87.9	N/A	N/A
5.12	1 of 1	4.624	90.3	N/A	N/A
12.80	1 of 1	12.255	95.7	N/A	N/A
32.00	1 of 1	30.748	96.1	N/A	N/A
80.00	1 of 1	78.047	97.6	N/A	N/A
200.00	1 of 1	204.571	102.3	N/A	N/A

Analyte Name: Luteolin

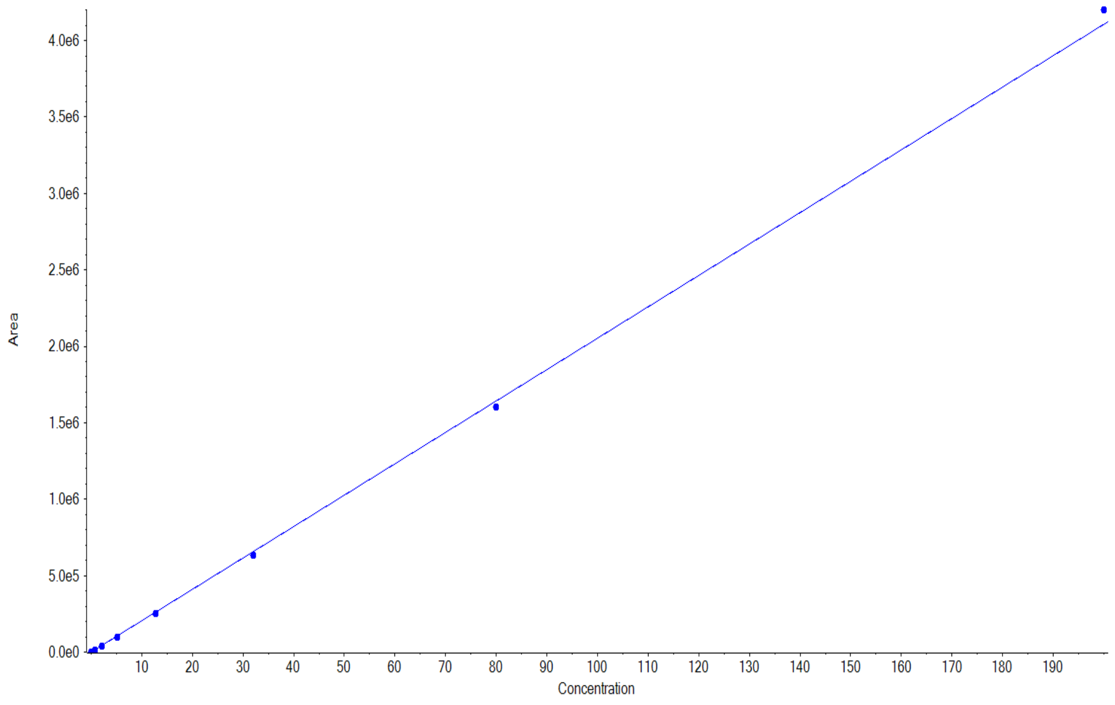

Analyte Name: Morin

Internal Standard: N/A

**Data File**	81089-BLK.wiff	**Result Table**	DZLM2023080667-results-20230907-5500(mix129)
**Acquisition Date**	8/21/2023 1:07:38 PM	**Algorithm Used**	MQ4
**Acquisition Method**	20230306-flavones(mix130-T3)-15min.dam	**Instrument Name**	Triple Quad 5500+
**Project**	N/A	**Processing Method**	20230329-flavones-(mix130-T3)-15min.qmethod

Regression Equation: y = 3.36826e4 x + -5814.08767 (r = 0.99974, r^2 = 0.99948)

(weighting: 1 / x)

Expected Concentration	Number of Values	Mean Calculated Concentration (ng/mL)	% Accuracy	Std. Deviation	%CV
0.02	0 of 1	N/A	N/A	N/A	N/A
0.05	0 of 1	N/A	N/A	N/A	N/A
0.13	0 of 1	N/A	N/A	N/A	N/A
0.33	1 of 1	0.403	122.2	N/A	N/A
0.82	1 of 1	0.833	101.6	N/A	N/A
2.05	1 of 1	1.926	93.9	N/A	N/A
5.12	1 of 1	4.432	86.6	N/A	N/A
12.80	1 of 1	12.226	95.5	N/A	N/A
32.00	1 of 1	31.511	98.5	N/A	N/A
80.00	1 of 1	81.069	101.3	N/A	N/A
200.00	1 of 1	200.719	100.4	N/A	N/A

Analyte Name: Morin

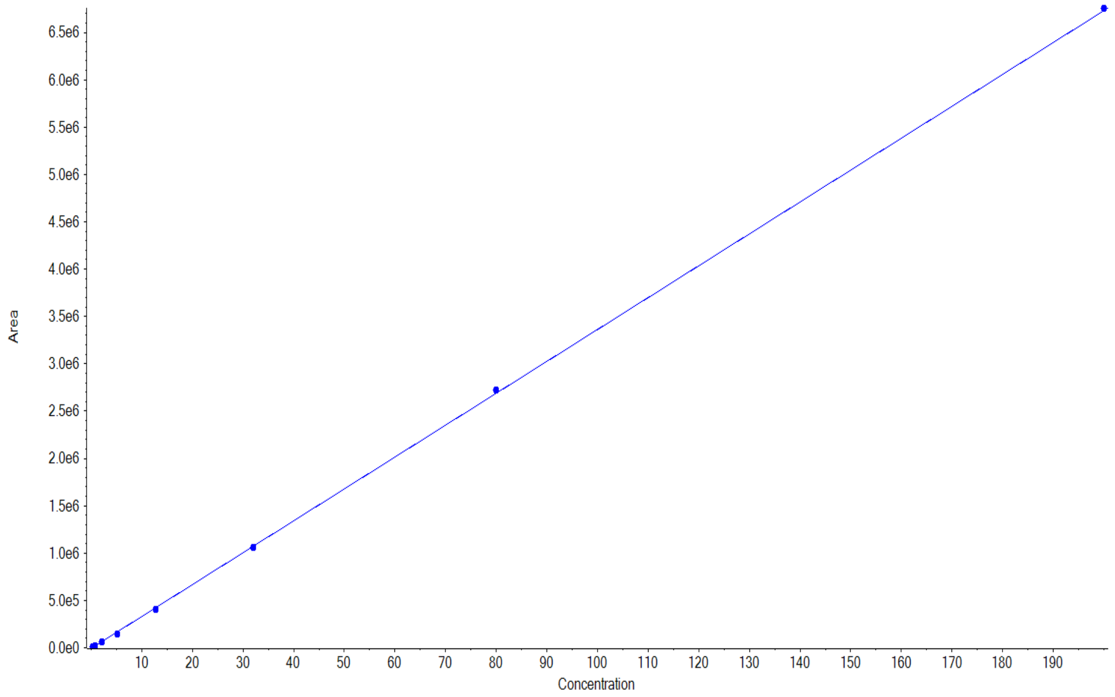

22

Analyte Name: Naringenin

Internal Standard: N/A

**Data File**	81089-BLK.wiff	**Result Table**	DZLM2023080667-results-20230907-5500(mix129)
**Acquisition Date**	8/21/2023 1:07:38 PM	**Algorithm Used**	MQ4
**Acquisition Method**	20230306-flavones(mix130-T3)-15min.dam	**Instrument Name**	Triple Quad 5500+
**Project**	N/A	**Processing Method**	20230329-flavones-(mix130 15min.qmethod

Regression Equation: y = 5528.47686 x + 262.23203 (r = 0.99972, r² = 0.99944)

(weighting: 1 / x)

Expected Concentration	Number of Values	Mean Calculated Concentration (ng/mL)	% Accuracy	Std. Deviation	%CV
0.02	0 of 1	N/A	N/A	N/A	N/A
0.05	0 of 1	N/A	N/A	N/A	N/A
0.13	0 of 1	N/A	N/A	N/A	N/A
0.33	1 of 1	0.346	104.7	N/A	N/A
0.82	1 of 1	0.835	101.9	N/A	N/A
2.05	1 of 1	2.159	105.3	N/A	N/A
5.12	1 of 1	4.743	92.6	N/A	N/A
12.80	1 of 1	12.153	94.9	N/A	N/A
32.00	1 of 1	31.278	97.7	N/A	N/A
80.00	1 of 1	82.675	103.3	N/A	N/A
200.00	1 of 1	198.933	99.5	N/A	N/A

Analyte Name: Naringenin

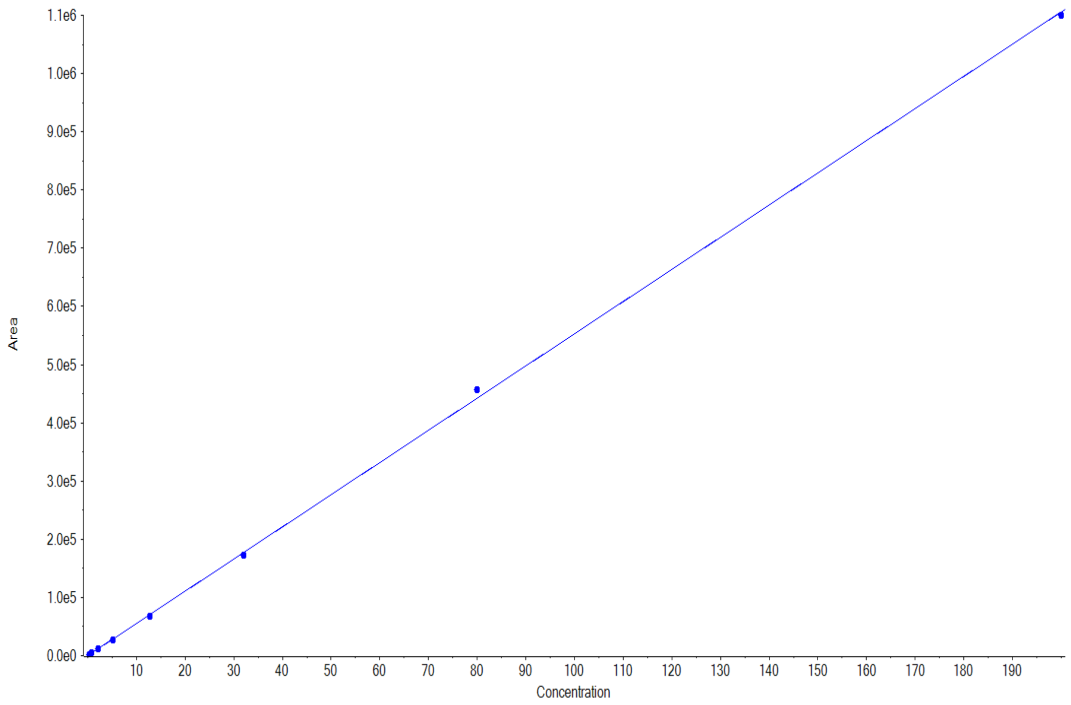

23

Analyte Name: Nobiletin

Internal Standard: N/A

**Data File**	81089-BLK.wiff	**Result Table**	DZLM2023080667-results-20230907-5500(mix129)
**Acquisition Date**	8/21/2023 1:07:38 PM	**Algorithm Used**	MQ4
**Acquisition Method**	20230306-flavones(mix130-T3)-15min.dam	**Instrument Name**	Triple Quad 5500+
**Project**	N/A	**Processing Method**	20230329-flavones-(mix130-T3)-15min.qmethod

Regression Equation: y = 1.15646e5 x + 259.95692 (r = 0.99926, r^2 = 0.99851)

(weighting: 1 / x)

Expected Concentration	Number of Values	Mean Calculated Concentration (ng/mL)	% Accuracy	Std. Deviation	%CV
0.02	1 of 1	0.022	111.1	N/A	N/A
0.05	1 of 1	0.048	95.1	N/A	N/A
0.13	1 of 1	0.130	99.9	N/A	N/A
0.33	1 of 1	0.296	89.7	N/A	N/A
0.82	1 of 1	0.733	89.4	N/A	N/A
2.05	1 of 1	2.093	102.1	N/A	N/A
5.12	1 of 1	5.382	105.1	N/A	N/A
12.80	1 of 1	13.640	106.6	N/A	N/A
32.00	1 of 1	33.258	103.9	N/A	N/A
80.00	1 of 1	77.719	97.2	N/A	N/A
200.00	0 of 1	N/A	N/A	N/A	N/A

Analyte Name: Nobiletin

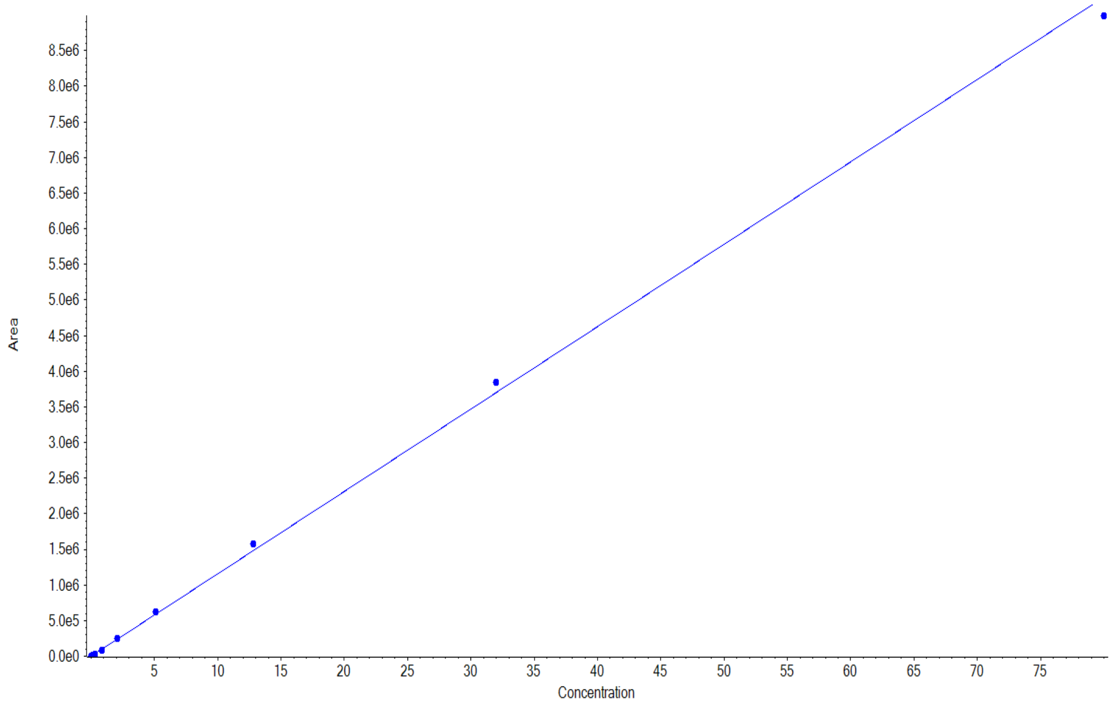

24

Analyte Name: Procyanidin B3

Internal Standard: N/A

**Data File**	81089-BLK.wiff	**Result Table**	DZLM2023080667-results-20230907-5500(mix129)
**Acquisition Date**	8/21/2023 1:07:38 PM	**Algorithm Used**	MQ4
**Acquisition Method**	20230306-flavones(mix130-T3)-15min.dam	**Instrument Name**	Triple Quad 5500+
**Project**	N/A	**Processing Method**	20230329-flavones-(mix130-T3)-15min.qmethod

Regression Equation: y = 1027.86158 x + -618.76208 (r = 0.99965, r^2 = 0.99930)

(weighting: 1 / x)

Expected Concentration	Number of Values	Mean Calculated Concentration (ng/mL)	% Accuracy	Std. Deviation	%CV
0.02	0 of 1	N/A	N/A	N/A	N/A
0.05	0 of 1	N/A	N/A	N/A	N/A
0.13	0 of 1	N/A	N/A	N/A	N/A
0.33	0 of 1	N/A	N/A	N/A	N/A
0.82	0 of 1	N/A	N/A	N/A	N/A
2.05	1 of 1	1.761	85.9	N/A	N/A
5.12	1 of 1	5.557	108.5	N/A	N/A
12.80	1 of 1	12.941	101.1	N/A	N/A
32.00	1 of 1	33.829	105.7	N/A	N/A
80.00	1 of 1	79.731	99.7	N/A	N/A
200.00	1 of 1	198.151	99.1	N/A	N/A

Analyte Name: Procyanidin B3

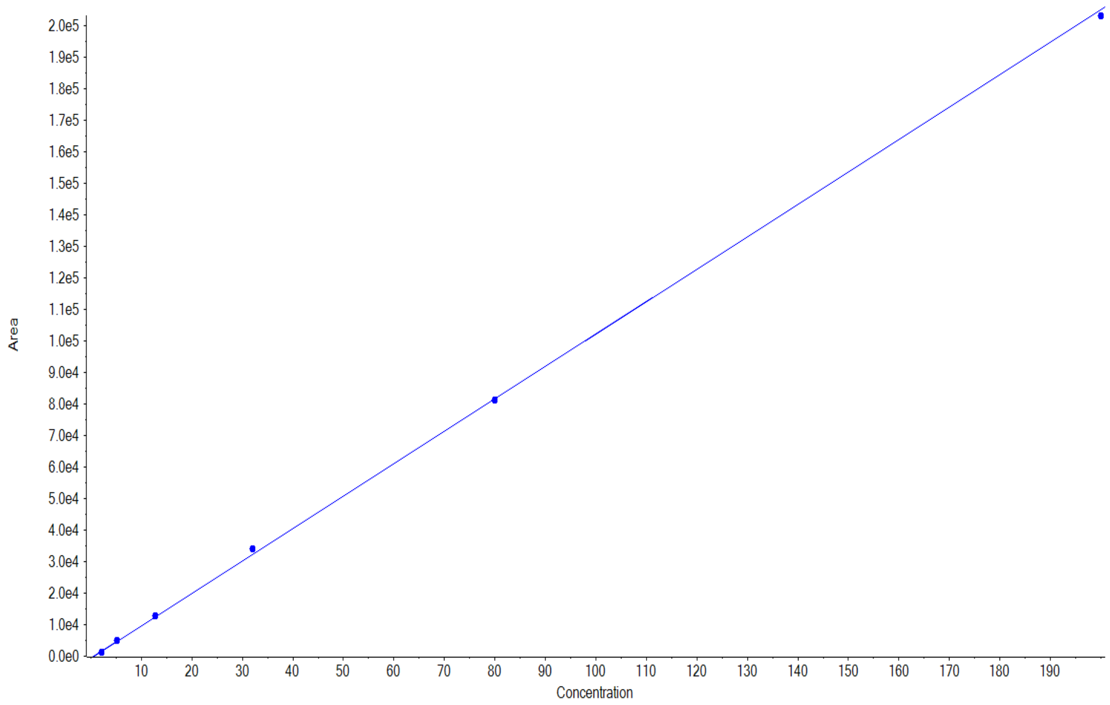

Analyte Name: Protocatechuic acid

Internal Standard: N/A

**Data File**	81089-BLK.wiff	**Result Table**	DZLM2023080667-results-20230907-5500(mix129)
**Acquisition Date**	8/21/2023 1:07:38 PM	**Algorithm Used**	MQ4
**Acquisition Method**	20230306-flavones(mix130-T3)-15min.dam	**Instrument Name**	Triple Quad 5500+
**Project**	N/A	**Processing Method**	20230329-flavones-(mix130-T3)-15min.qmethod

Regression Equation: y = 28649.61951 x + 5.11337e4 (r = 0.99996, r^2 = 0.99992)

(weighting: 1 / x)

Expected Concentration	Number of Values	Mean Calculated Concentration (ng/mL)	% Accuracy	Std. Deviation	%CV
0.02	0 of 1	N/A	N/A	N/A	N/A
0.05	0 of 1	N/A	N/A	N/A	N/A
0.13	0 of 1	N/A	N/A	N/A	N/A
0.33	0 of 1	N/A	N/A	N/A	N/A
0.82	1 of 1	0.825	100.6	N/A	N/A
2.05	1 of 1	2.045	99.8	N/A	N/A
5.12	1 of 1	5.052	98.7	N/A	N/A
12.80	1 of 1	12.961	101.3	N/A	N/A
32.00	1 of 1	31.907	99.7	N/A	N/A
80.00	0 of 1	N/A	N/A	N/A	N/A
200.00	0 of 1	N/A	N/A	N/A	N/A

Analyte Name: Protocatechuic acid

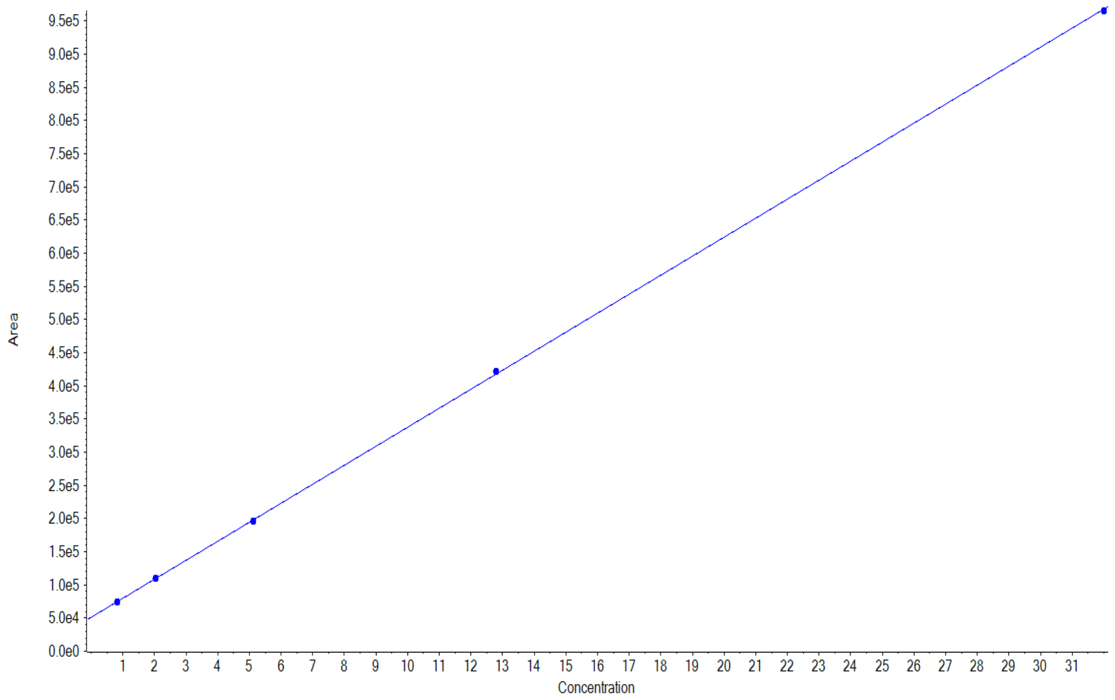

Analyte Name: Quercetin

Internal Standard: N/A

**Data File**	81089-BLK.wiff	**Result Table**	DZLM2023080667-results-20230907-5500(mix129)
**Acquisition Date**	8/21/2023 1:07:38 PM	**Algorithm Used**	MQ4
**Acquisition Method**	20230306-flavones(mix130-T3)-15min.dam	**Instrument Name**	Triple Quad 5500+
**Project**	N/A	**Processing Method**	20230329-flavones-(mix130-T3)-15min.qmethod

Regression Equation: y = 3.57750e4 x + -2174.81047 (r = 0.99953, r^2 = 0.99907)

(weighting: 1 / x)

Expected Concentration	Number of Values	Mean Calculated Concentration (ng/mL)	% Accuracy	Std. Deviation	%CV
0.02	0 of 1	N/A	N/A	N/A	N/A
0.05	1 of 1	0.076	153.0	N/A	N/A
0.13	0 of 1	N/A	N/A	N/A	N/A
0.33	0 of 1	N/A	N/A	N/A	N/A
0.82	1 of 1	0.629	76.7	N/A	N/A
2.05	1 of 1	1.825	89.1	N/A	N/A
5.12	1 of 1	4.521	88.3	N/A	N/A
12.80	1 of 1	11.557	90.3	N/A	N/A
32.00	1 of 1	32.696	102.2	N/A	N/A
80.00	1 of 1	79.669	99.6	N/A	N/A
200.00	1 of 1	201.866	100.9	N/A	N/A

Analyte Name: Quercetin

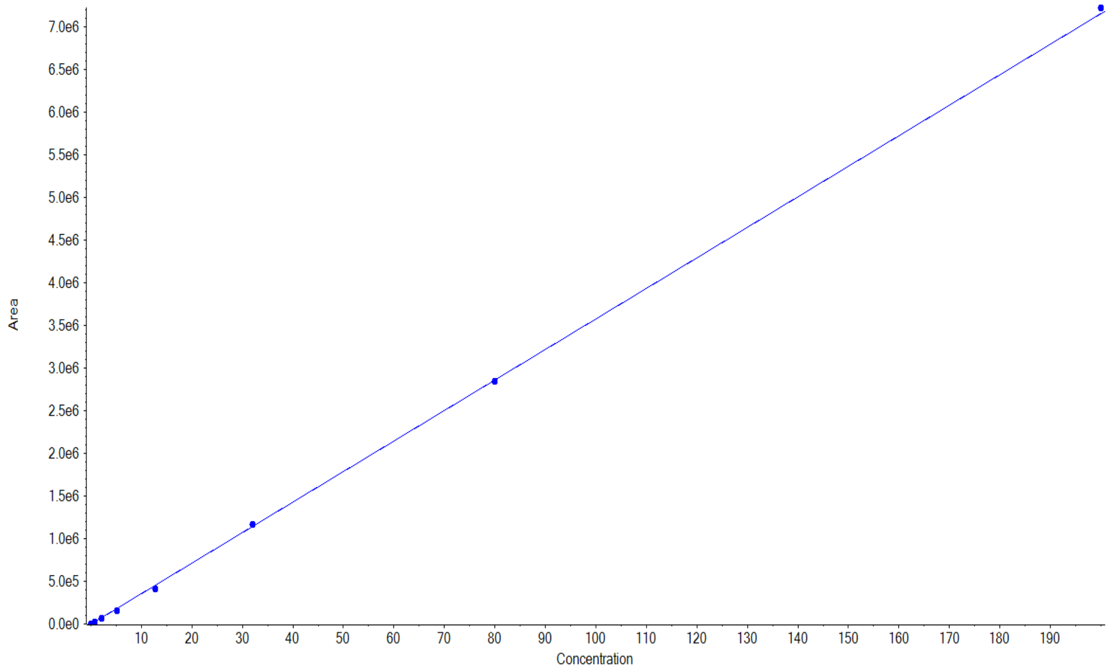

27

Analyte Name: Quercetin 3-galactoside

Internal Standard: N/A

**Data File**	81089-BLK.wiff	**Result Table**	DZLM2023080667-results-20230907-5500(mix129)
**Acquisition Date**	8/21/2023 1:07:38 PM	**Algorithm Used**	MQ4
**Acquisition Method**	20230306-flavones(mix130-T3)-15min.dam	**Instrument Name**	Triple Quad 5500+
**Project**	N/A	**Processing Method**	20230329-flavones-(mix130-T3)-15min.qmethod

Regression Equation: y = 5392.00017 x + -725.14279 (r = 0.99911, r^2 = 0.99822)

(weighting: 1 / x)

Expected Concentration	Number of Values	Mean Calculated Concentration (ng/mL)	% Accuracy	Std. Deviation	%CV
0.02	0 of 1	N/A	N/A	N/A	N/A
0.05	0 of 1	N/A	N/A	N/A	N/A
0.13	0 of 1	N/A	N/A	N/A	N/A
0.33	0 of 1	N/A	N/A	N/A	N/A
0.82	1 of 1	1.025	125.0	N/A	N/A
2.05	1 of 1	1.669	81.4	N/A	N/A
5.12	1 of 1	4.564	89.1	N/A	N/A
12.80	1 of 1	12.768	99.8	N/A	N/A
32.00	1 of 1	32.295	100.9	N/A	N/A
80.00	1 of 1	84.694	105.9	N/A	N/A
200.00	1 of 1	195.775	97.9	N/A	N/A

Analyte Name: Quercetin 3-galactoside

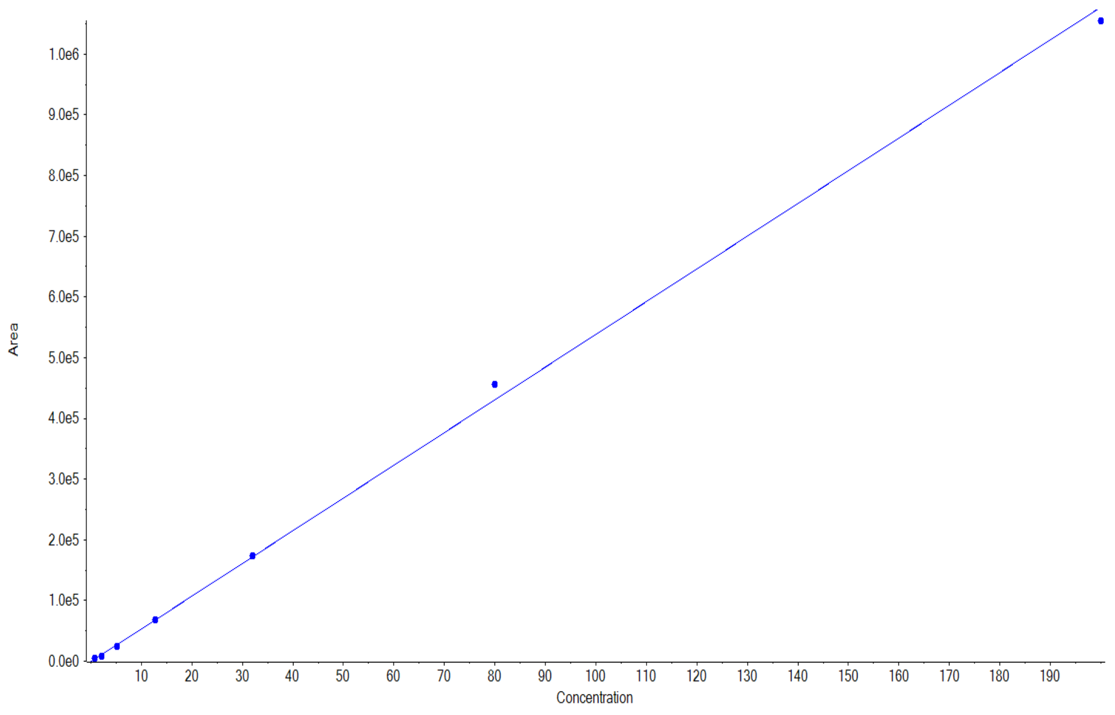

Analyte Name: Rutin

Internal Standard: N/A

**Data File**	81089-BLK.wiff	**Result Table**	DZLM2023080667-results-20230907-5500(mix129)
**Acquisition Date**	8/21/2023 1:07:38 PM	**Algorithm Used**	MQ4
**Acquisition Method**	20230306-flavones(mix130-T3)-15min.dam	**Instrument Name**	Triple Quad 5500+
**Project**	N/A	**Processing Method**	20230329-flavones-(mix130-T3)-15min.qmethod

Regression Equation: y = 5036.91053 x + 3683.16333 (r = 0.99949, r^2 = 0.99898)

(weighting: 1 / x)

Expected Concentration	Number of Values	Mean Calculated Concentration (ng/mL)	% Accuracy	Std. Deviation	%CV
0.02	0 of 1	N/A	N/A	N/A	N/A
0.05	0 of 1	N/A	N/A	N/A	N/A
0.13	0 of 1	N/A	N/A	N/A	N/A
0.33	0 of 1	N/A	N/A	N/A	N/A
0.82	0 of 1	N/A	N/A	N/A	N/A
2.05	1 of 1	1.908	93.1	N/A	N/A
5.12	1 of 1	5.587	109.1	N/A	N/A
12.80	1 of 1	11.743	91.7	N/A	N/A
32.00	1 of 1	34.137	106.7	N/A	N/A
80.00	1 of 1	80.087	100.1	N/A	N/A
200.00	1 of 1	198.508	99.3	N/A	N/A

Analyte Name: Rutin

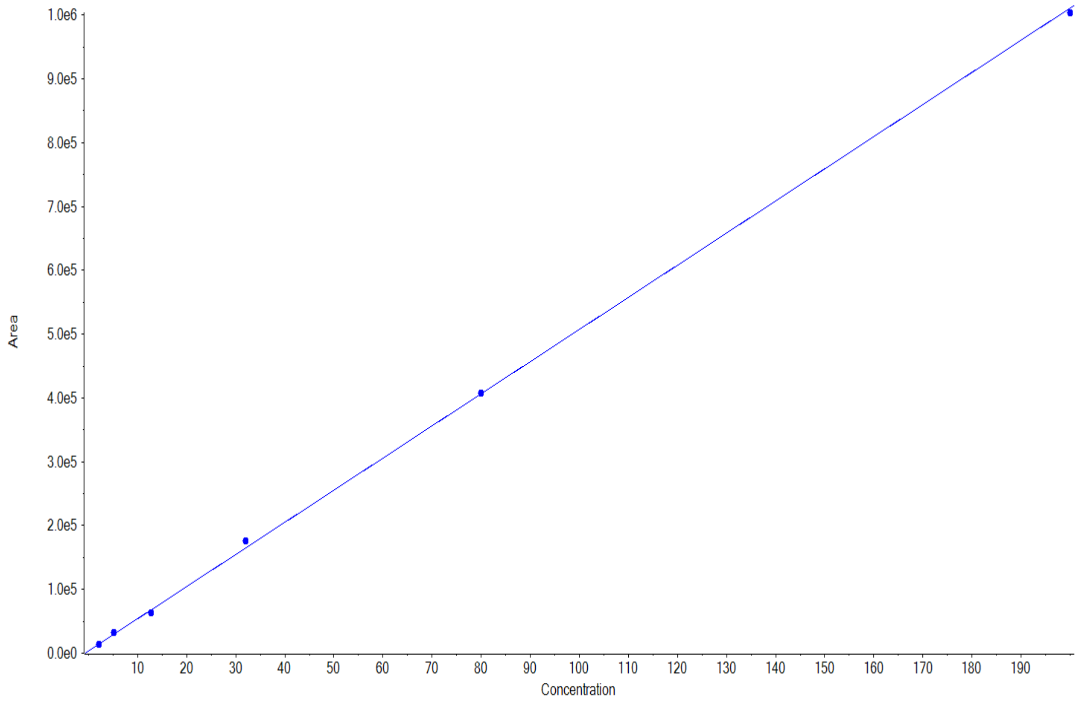

29

Analyte Name: Salicylic acid

Internal Standard: N/A

**Data File**	81089-BLK.wiff	**Result Table**	DZLM2023080667-results-20230907-5500(mix129)
**Acquisition Date**	8/21/2023 1:07:38 PM	**Algorithm Used**	MQ4
**Acquisition Method**	20230306-flavones(mix130-T3)-15min.dam	**Instrument Name**	Triple Quad 5500+
**Project**	N/A	**Processing Method**	20230329-flavones-(mix130-T3)-15min.qmethod

Regression Equation: y = 7.20976e4 x + -4003.94366 (r = 0.99987, r^2 = 0.99974)

(weighting: 1 / x)

Expected Concentration	Number of Values	Mean Calculated Concentration (ng/mL)	% Accuracy	Std. Deviation	%CV
0.02	0 of 1	N/A	N/A	N/A	N/A
0.05	0 of 1	N/A	N/A	N/A	N/A
0.13	0 of 1	N/A	N/A	N/A	N/A
0.33	0 of 1	N/A	N/A	N/A	N/A
0.82	1 of 1	0.887	108.2	N/A	N/A
2.05	1 of 1	2.004	97.8	N/A	N/A
5.12	1 of 1	5.049	98.6	N/A	N/A
12.80	1 of 1	12.480	97.5	N/A	N/A
32.00	1 of 1	30.786	96.2	N/A	N/A
80.00	1 of 1	81.210	101.5	N/A	N/A
200.00	1 of 1	200.374	100.2	N/A	N/A

Analyte Name: Salicylic acid

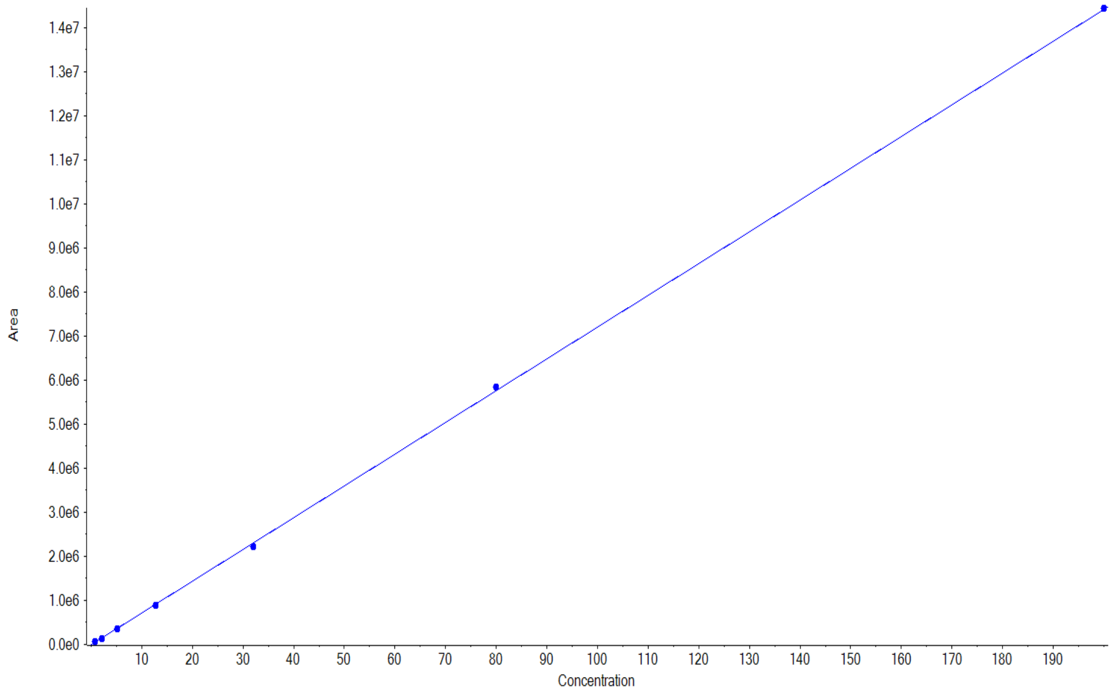

30

Analyte Name: Syringic acid

Internal Standard: N/A

**Data File**	81089-BLK.wiff	**Result Table**	DZLM2023080667-results-20230907-5500(mix129)
**Acquisition Date**	8/21/2023 1:07:38 PM	**Algorithm Used**	MQ4
**Acquisition Method**	20230306-flavones(mix130-T3)-15min.dam	**Instrument Name**	Triple Quad 5500+
**Project**	N/A	**Processing Method**	20230329-flavones-(mix130-T3)-15min.qmethod

Regression Equation: y = 7290.34988 x + 20593.33347 (r = 0.99848, r^2 = 0.99696)

(weighting: 1 / x)

Expected Concentration	Number of Values	Mean Calculated Concentration (ng/mL)	% Accuracy	Std. Deviation	%CV
0.02	0 of 1	N/A	N/A	N/A	N/A
0.05	0 of 1	N/A	N/A	N/A	N/A
0.13	0 of 1	N/A	N/A	N/A	N/A
0.33	0 of 1	N/A	N/A	N/A	N/A
0.82	0 of 1	N/A	N/A	N/A	N/A
2.05	1 of 1	2.245	109.5	N/A	N/A
5.12	1 of 1	4.517	88.2	N/A	N/A
12.80	1 of 1	12.389	96.8	N/A	N/A
32.00	1 of 1	34.367	107.4	N/A	N/A
80.00	1 of 1	78.451	98.1	N/A	N/A
200.00	0 of 1	N/A	N/A	N/A	N/A

Analyte Name: Syringic acid

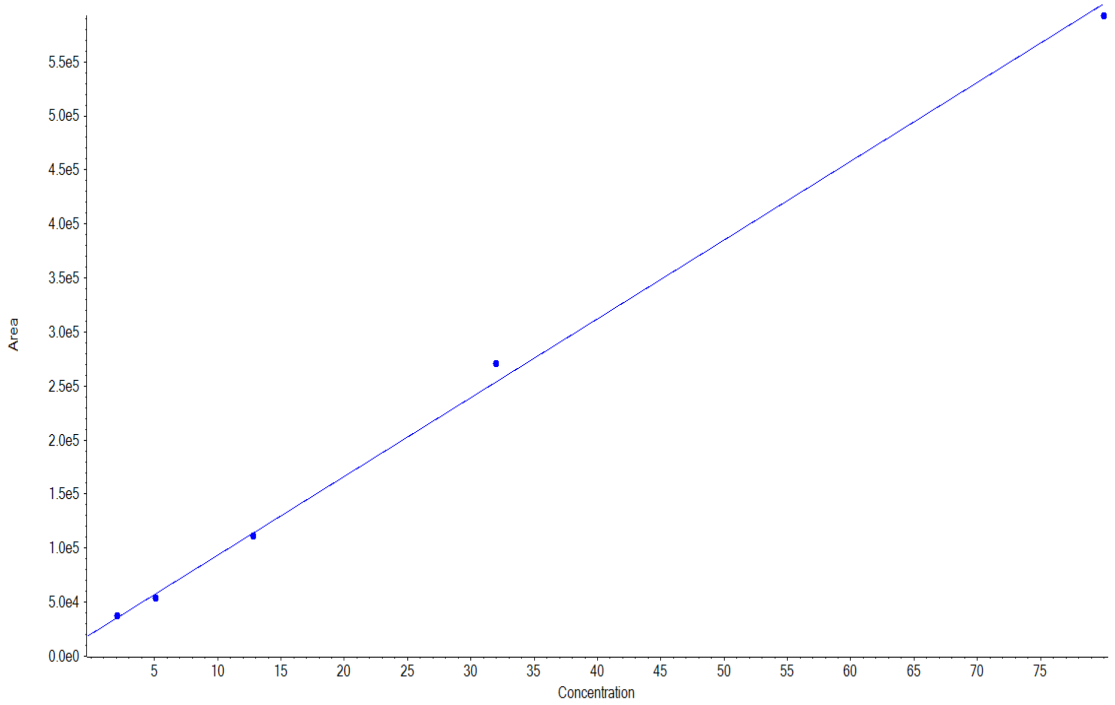

31

Analyte Name: trans-Cinnamic acid

Internal Standard: N/A

**Data File**	81089-BLK.wiff	**Result Table**	DZLM2023080667-results-20230907-5500(mix129)
**Acquisition Date**	8/21/2023 1:07:38 PM	**Algorithm Used**	MQ4
**Acquisition Method**	20230306-flavones(mix130-T3)-15min.dam	**Instrument Name**	Triple Quad 5500+
**Project**	N/A	**Processing Method**	20230329-flavones-(mix130-T3)-15min.qmethod

Regression Equation: y = 9178.47821 x + 20318.72051 (r = 0.99950, r² = 0.99900)

(weighting: 1 / x)

Expected Concentration	Number of Values	Mean Calculated Concentration (ng/mL)	% Accuracy	Std. Deviation	%CV
0.02	0 of 1	N/A	N/A	N/A	N/A
0.05	0 of 1	N/A	N/A	N/A	N/A
0.13	0 of 1	N/A	N/A	N/A	N/A
0.33	0 of 1	N/A	N/A	N/A	N/A
0.82	0 of 1	N/A	N/A	N/A	N/A
2.05	0 of 1	N/A	N/A	N/A	N/A
5.12	1 of 1	4.646	90.7	N/A	N/A
12.80	1 of 1	13.251	103.5	N/A	N/A
32.00	1 of 1	33.627	105.1	N/A	N/A
80.00	1 of 1	81.937	102.4	N/A	N/A
200.00	1 of 1	196.459	98.2	N/A	N/A

Analyte Name: trans-Cinnamic acid

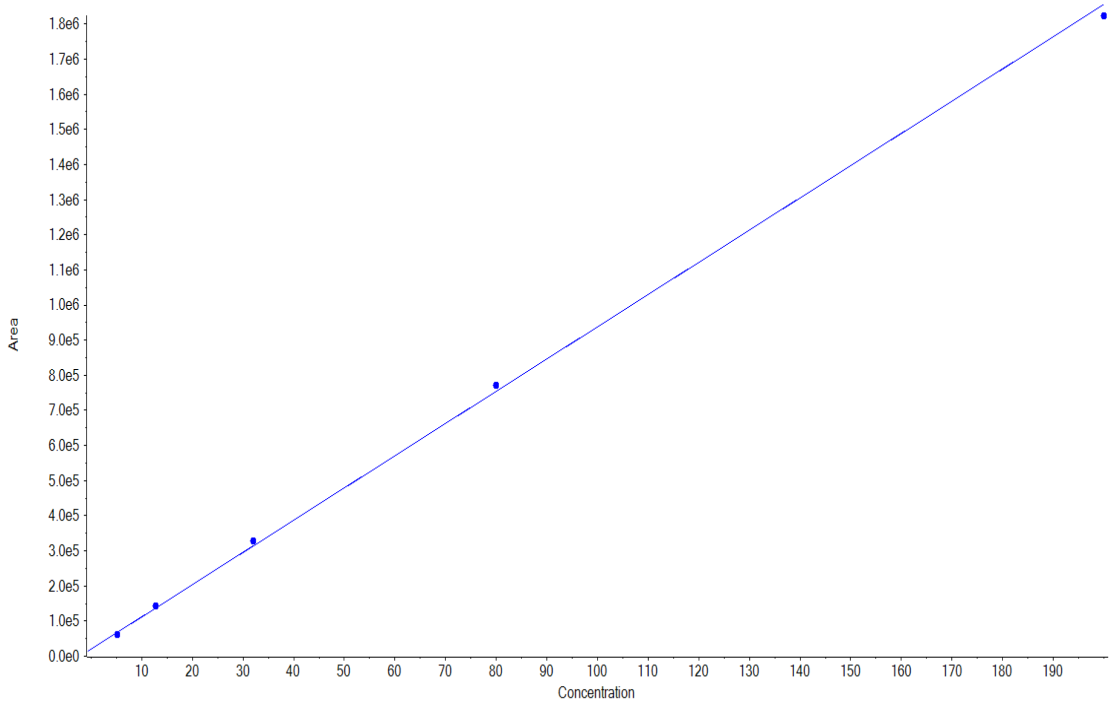

Analyte Name: Umbelliferone

Internal Standard: N/A

**Data File**	81089-BLK.wiff	**Result Table**	DZLM2023080667-results-20230907-5500(mix129)
**Acquisition Date**	8/21/2023 1:07:38 PM	**Algorithm Used**	MQ4
**Acquisition Method**	20230306-flavones(mix130-T3)-15min.dam	**Instrument Name**	Triple Quad 5500+
**Project**	N/A	**Processing Method**	20230329-flavones-(mix130-T3)-15min.qmethod

Regression Equation: y = 27037.41986 x + 18002.73471 (r = 0.99894, r² = 0.99788) (weighting: 1 / x)

Expected Concentration	Number of Values	Mean Calculated Concentration (ng/mL)	% Accuracy	Std. Deviation	%CV
0.02	0 of 1	N/A	N/A	N/A	N/A
0.05	0 of 1	N/A	N/A	N/A	N/A
0.13	0 of 1	N/A	N/A	N/A	N/A
0.33	0 of 1	N/A	N/A	N/A	N/A
0.82	0 of 1	N/A	N/A	N/A	N/A
2.05	1 of 1	2.102	102.5	N/A	N/A
5.12	1 of 1	4.546	88.8	N/A	N/A
12.80	1 of 1	12.491	97.6	N/A	N/A
32.00	1 of 1	35.512	111.0	N/A	N/A
80.00	1 of 1	81.924	102.4	N/A	N/A
200.00	1 of 1	195.395	97.7	N/A	N/A

Analyte Name: Umbelliferone

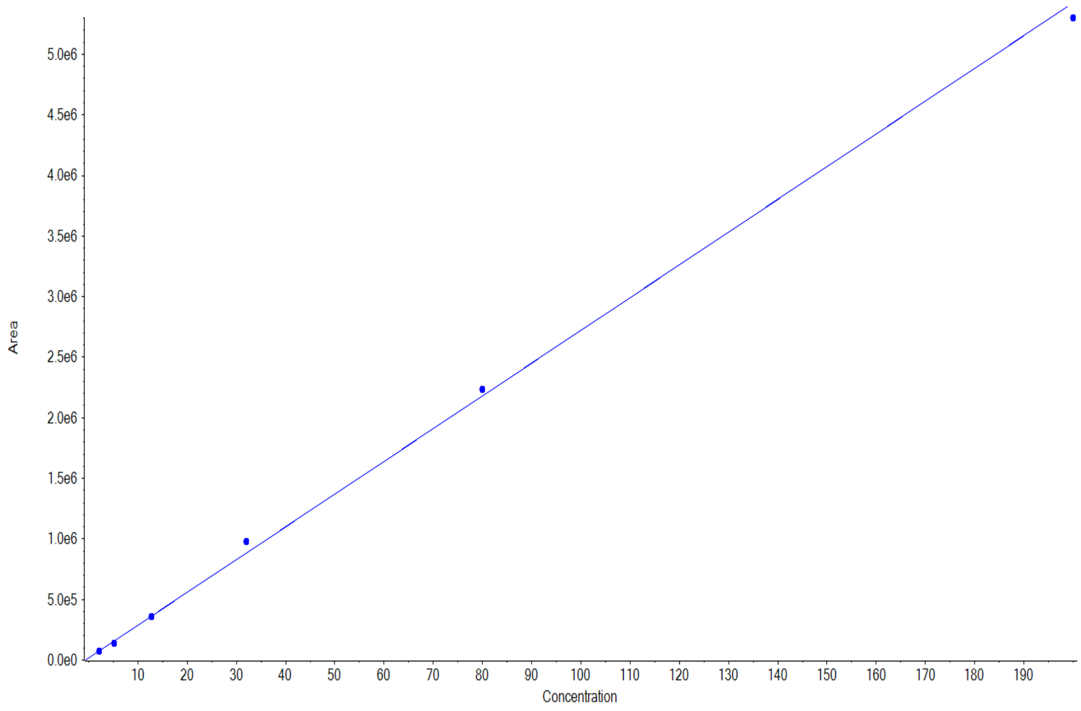

Analyte Name: Vanillic acid

Internal Standard: N/A

**Data File**	81089-BLK.wiff	**Result Table**	DZLM2023080667-results-20230907-5500(mix129)
**Acquisition Date**	8/21/2023 1:07:38 PM	**Algorithm Used**	MQ4
**Acquisition Method**	20230306-flavones(mix130-T3)-15min.dam	**Instrument Name**	Triple Quad 5500+
**Project**	N/A	**Processing Method**	20230329-flavones-(mix130-T3)-15min.qmethod

Regression Equation: y = 244.39591 x + 618.36807 (r = 0.99803, r^2 = 0.99607)

(weighting: 1 / x)

Expected Concentration	Number of Values	Mean Calculated Concentration (ng/mL)	% Accuracy	Std. Deviation	%CV
0.02	0 of 1	N/A	N/A	N/A	N/A
0.05	0 of 1	N/A	N/A	N/A	N/A
0.13	0 of 1	N/A	N/A	N/A	N/A
0.33	0 of 1	N/A	N/A	N/A	N/A
0.82	1 of 1	0.816	99.5	N/A	N/A
2.05	1 of 1	1.895	92.4	N/A	N/A
5.12	1 of 1	5.759	112.5	N/A	N/A
12.80	1 of 1	12.952	101.2	N/A	N/A
32.00	1 of 1	28.054	87.7	N/A	N/A
80.00	1 of 1	86.793	108.5	N/A	N/A
200.00	1 of 1	196.521	98.3	N/A	N/A

Analyte Name: Vanillic acid

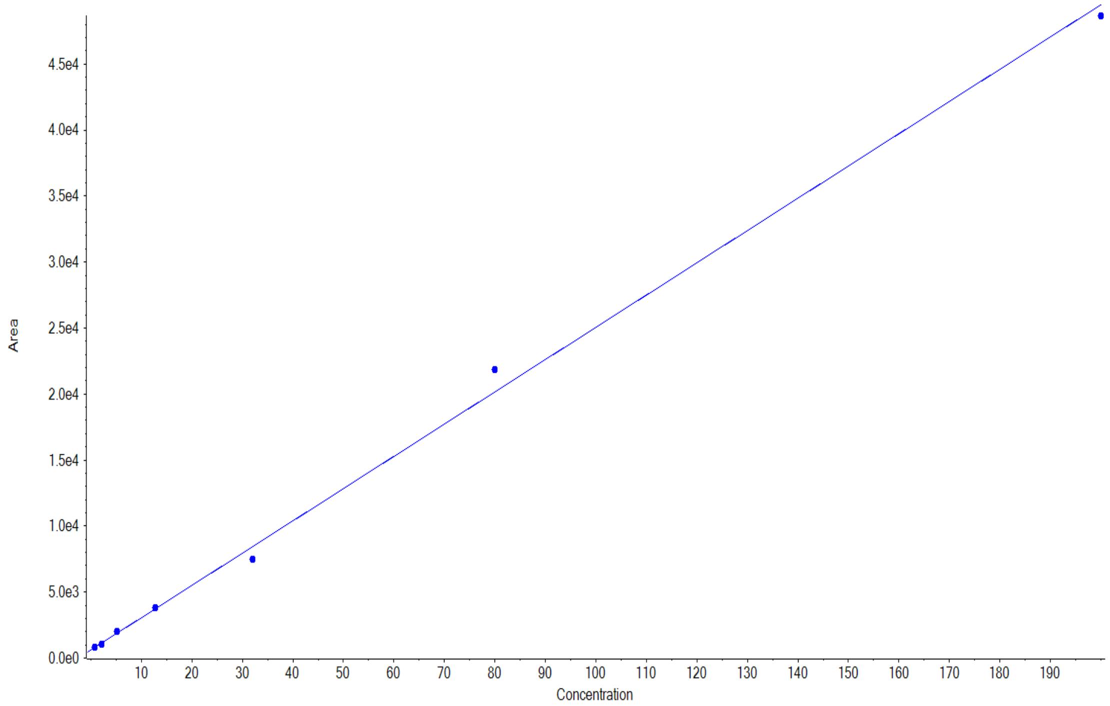

34

Analyte Name: Vitexin

Internal Standard: N/A

**Data File**	81089-BLK.wiff	**Result Table**	DZLM2023080667-results-20230907-5500(mix129)
**Acquisition Date**	8/21/2023 1:07:38 PM	**Algorithm Used**	MQ4
**Acquisition Method**	20230306-flavones(mix130-T3)-15min.dam	**Instrument Name**	Triple Quad 5500+
**Project**	N/A	**Processing Method**	20230329-flavones-(mix130-T3)-15min.qmethod

Regression Equation: y = 16218.98703 x + 1713.72928 (r = 0.99977, r² = 0.99954)

(weighting: 1 / x)

Expected Concentration	Number of Values	Mean Calculated Concentration (ng/mL)	% Accuracy	Std. Deviation	%CV
0.02	0 of 1	N/A	N/A	N/A	N/A
0.05	0 of 1	N/A	N/A	N/A	N/A
0.13	0 of 1	N/A	N/A	N/A	N/A
0.33	1 of 1	0.290	87.8	N/A	N/A
0.82	1 of 1	0.906	110.5	N/A	N/A
2.05	1 of 1	2.233	108.9	N/A	N/A
5.12	1 of 1	4.820	94.1	N/A	N/A
12.80	1 of 1	12.506	97.7	N/A	N/A
32.00	1 of 1	32.268	100.8	N/A	N/A
80.00	1 of 1	80.097	100.1	N/A	N/A
200.00	0 of 1	N/A	N/A	N/A	N/A

Analyte Name: Vitexin

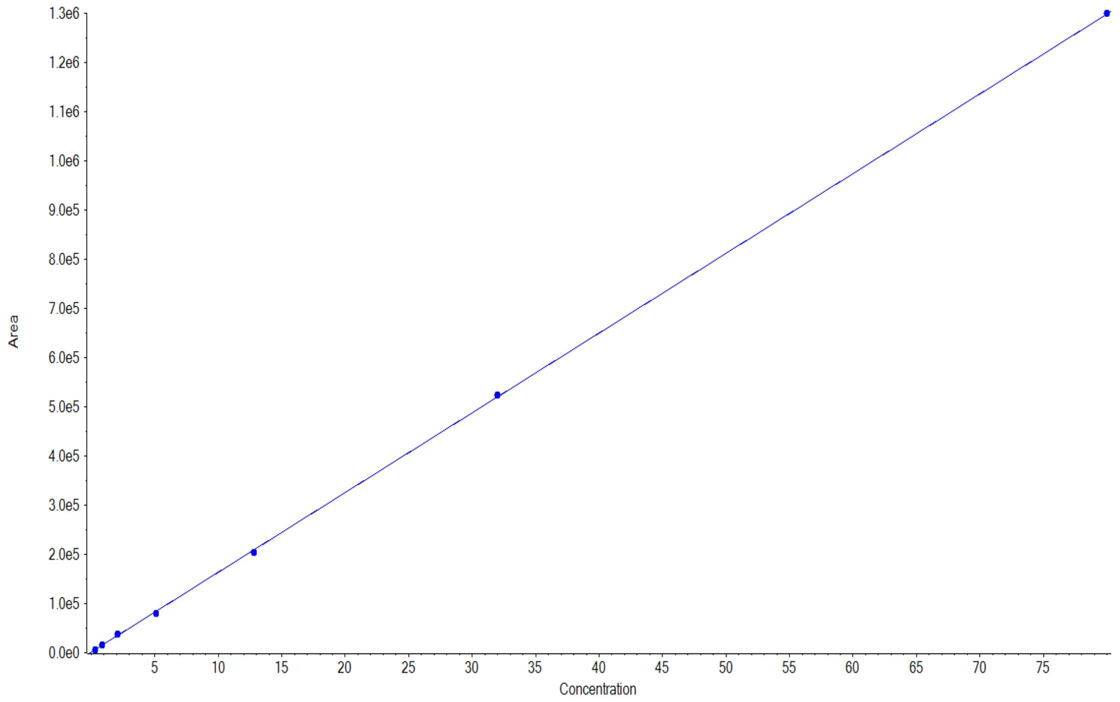

Supplement: Supplementary file 1 [file antioxidants-13-00620-s001.zip › Table S1.pdf]
