# Supplementary figures and images for: Metabolomic Profiling of Floccularia luteovirens from Different Geographical Regions Proposes a Novel Perspective on Their Antioxidative Activities
Source: Antioxidants (Basel). 2024 May 20;13(5):620. doi: 10.3390/antiox13050620 (PMC11118160; doi:10.3390/antiox13050620)

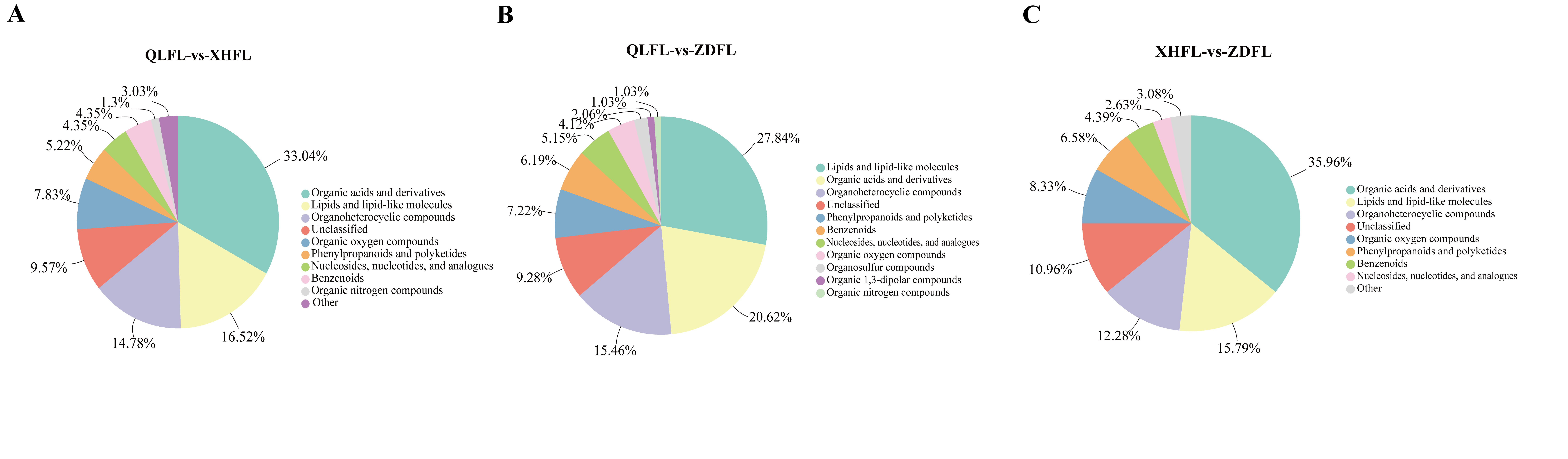

Supplement: Supplementary file 1 [file antioxidants-13-00620-s001.zip › Figure S1.tif]
